# Current Development of Geological Disposal Facilities: A Comprehensive Review of Corrosion and Microbially Influenced Corrosion of Nuclear Waste Canisters

**DOI:** 10.3390/ma19183873

**Published:** 2026-09-11

**Authors:** Adam D. Mumford, Yon Ju-Nam, Mohamed L. Merroun, Jesús J. Ojeda

**Affiliations:** 1 Department of Chemical Engineering, Faculty of Science and Engineering, Swansea University, Swansea SA1 8EN, UK; 962818@swansea.ac.uk (A.D.M.); y.k.ju-nam@swansea.ac.uk (Y.J.-N.); 2 Department of Microbiology, Faculty of Sciences, University of Granada, 18071 Granada, Spain; merroun@ugr.es

**Keywords:** corrosion, microbially influenced corrosion (MIC), nuclear waste, geological disposal facilities (GDFs), sulphate-reducing bacteria, material integrity

## Abstract

**Highlights:**

- Reviews corrosion of canister materials for nuclear waste isolation in GDFs.
- Highlights microbial threats to long-term canister integrity and durability.
- Discusses sulphate-reducing bacteria and their role in localised corrosion.
- Examines environmental factors driving corrosion over geological timescales.
- Identifies further research in MIC of canisters under repository-relevant conditions.

**Abstract:**

The long-term safety of geological disposal facilities (GDFs) depends in part on the integrity of metallic waste canisters exposed to evolving thermal, geochemical and microbial conditions. Although abiotic corrosion is comparatively well characterised, the significance of microbiologically influenced corrosion (MIC) remains uncertain. This review critically compares carbon steel, stainless steel, copper, and titanium under GDF-relevant conditions, distinguishing chemical MIC (CMIC) from electrical MIC (EMIC) and examining the roles of sulphate-reducing bacteria, biofilms, and extracellular electron transfer. Reported behaviour varies markedly: carbon-steel studies have reported localised attack approaching 1 mm within 12 months, while SRB-associated copper corrosion rates of up to 9.8 μm year^−1^ have been measured, yet other long-term experiments show little or no detectable microbial acceleration. These contrasts indicate that microbial presence alone is not predictive of corrosion severity; temperature, redox state, groundwater chemistry, bentonite density, nutrient availability, and passive-film stability are critical controls. The distinctive contribution of this review is an integrated, material-to-material assessment linking abiotic corrosion, CMIC and EMIC mechanisms with repository-specific environmental constraints, and current GDF development. It also identifies key uncertainties arising from methodological variability and short-term laboratory testing, supporting priorities for standardised, long-term, and in situ studies.

## 1. Introduction

Nuclear power remains an important low-carbon source of electricity, but its continued use and the decommissioning of existing reactors generate radioactive wastes that require safe long-term management [1,2,3]. High-level radioactive waste contains long-lived radionuclides that may require isolation for 100,000 years or more, making permanent disposal a major technical challenge. Geological disposal facilities (GDFs), also referred to as deep geological repositories, are internationally recognised as the preferred approach for the permanent disposal of high-level radioactive waste [4].

**Figure 1:**
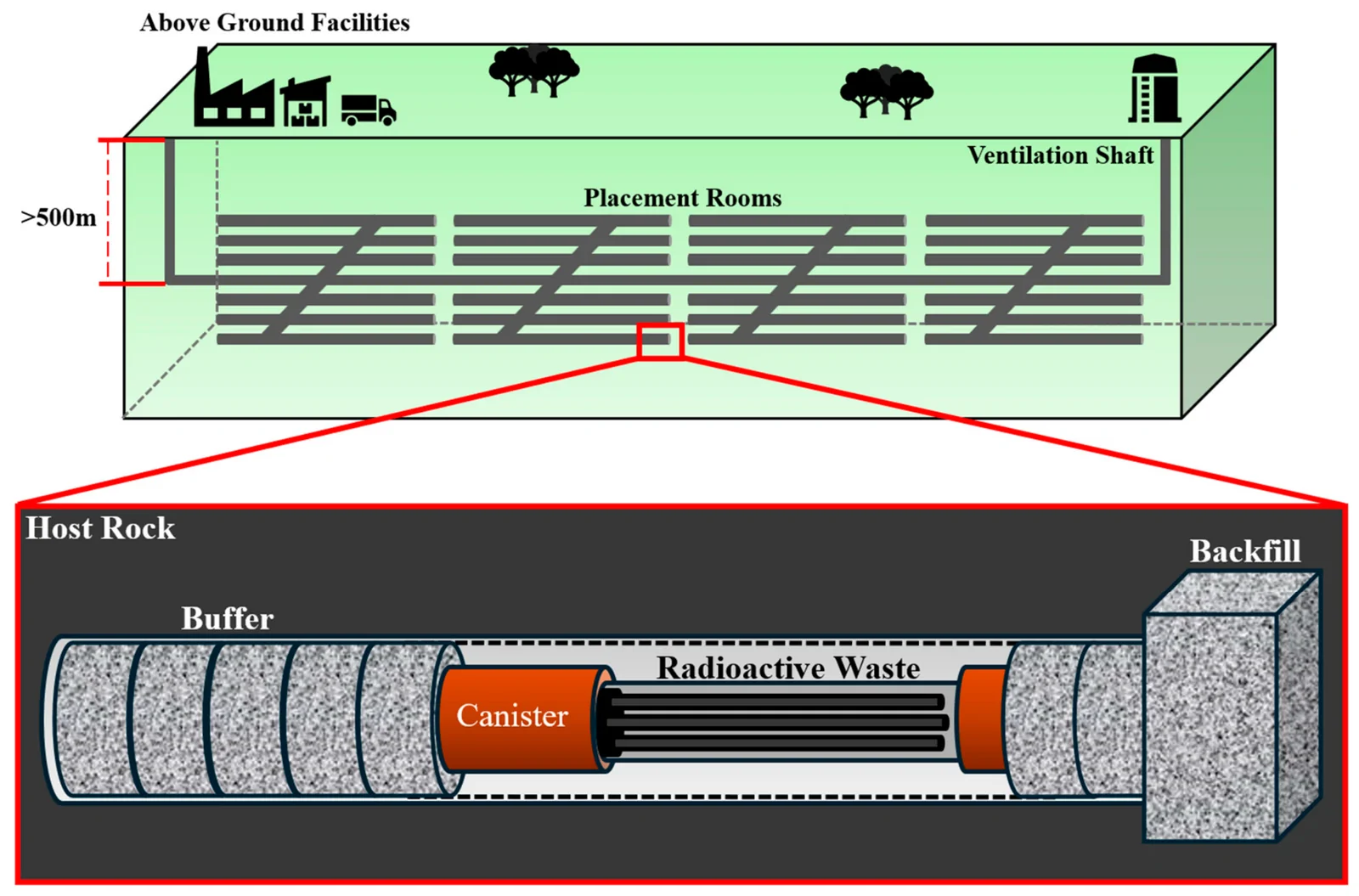
Typical GDF layout and barriers for HLW permanent disposal. Adapted from M. Ruiz-Fresneda, et al. (2023) [7] under the Creative Commons Attribution (CC BY 4.0) licence.

GDFs rely on a multi-barrier system combining natural geological formations with engineered barriers, including metallic canisters, buffer materials, and repository backfill, as illustrated in Figure 1 [5,6]. The metallic canister provides an important containment function but is exposed to evolving thermal, chemical, electrochemical, and microbiological conditions over repository timescales. Although the abiotic corrosion behaviour of candidate canister materials has been extensively investigated, greater uncertainty remains around microbiologically influenced corrosion (MIC), particularly the conditions under which microbial activity can alter local chemistry, destabilise passive films or promote localised corrosion.

This review evaluates the corrosion behaviour of the principal metallic canister materials, including carbon steel, stainless steel, copper, and titanium, with particular emphasis on MIC under GDF-relevant conditions. It compares material-specific degradation mechanisms, considers the roles of sulphate-reducing bacteria, biofilm development, and extracellular electron transfer, and assesses how repository conditions influence the significance of these processes. By integrating material-specific corrosion behaviour with microbial mechanisms and current repository conditions, this review aims to provide a comparative framework for identifying where MIC presents the greatest uncertainty for long-term canister performance. It also considers recent international developments in GDF implementation and identifies key limitations in the existing MIC literature, particularly the lack of long-term in situ data and standardised testing under repository-relevant conditions.

## 2. Classification of Radioactive Waste

According to the IAEA, the strategy for the management of radioactive waste varies depending on its classification, which is based on radionuclide inventory and half-life. Waste with a half-life of 31 years or less is considered short-lived, whereas waste exceeding this threshold is considered long-lived. The following categories have been established, as summarised in Table 1: high level waste (HLW), intermediate level waste (ILW), low level waste (LLW), very low level waste (VLLW), very short-lived waste (VSLW), and exempt waste (EW) [4]. However, classification criteria vary by country, and while the IAEA provides guidance, it does not enforce specific disposal regulations, leaving waste management decisions to national authorities.

As shown in Table 1, VSLW contains activity concentrations above clearance levels but has a short half-life (typically <100 days) [8,9]. This type of waste is primarily generated from medical diagnostics, research, and nuclear power operations. VSLW is temporarily stored, typically for <2 years, until its activity falls below regulatory limits, allowing for it to be reclassified as EW [8,14]. EW is considered non-radioactive waste because its radionuclide concentrations are below clearance levels, corresponding to an annual dose of 10 μSv or less [8,14].

VLLW arises predominantly from the decommissioning of nuclear facilities, mining, research, and medical applications, typically in the form of soil or rubble [4]. VLLW contains radionuclides with activity concentrations above clearance levels (~1 to 100 Bq/g). This waste does not require high-level containment and is usually stored above surface in engineered landfill-style facilities or near-surface repositories [4,8].

LLW is the most common form of waste by volume and has radionuclide content significantly above clearance levels, covering a wide concentration range. LLW is typically stored in near-surface disposal facilities (<10 m depth), with an engineered cap placed over disposal units to minimise water infiltration [8,16]. Some countries prefer to store LLW either in sub-surface facilities (below 10 metres underground), alongside ILW (~60 metres below ground) or in deeper repositories [4,8].

ILW requires greater containment and isolation than waste stored in surface disposal facilities, as it contains both short- and long-lived radionuclides [16]. Some ILW generates heat, requiring storage at depths ranging from tens to several hundred metres [16].

HLW contains the highest concentrations of radionuclides and generates significant radiogenic heat, which can persist for centuries to thousands of years [10]. HLW includes spent nuclear fuel and vitrified waste from fuel reprocessing [10]. Current regulations require HLW to be stored in geological and engineered barrier systems, typically in GDFs, which are internationally recognised as the preferred long-term disposal solution [4,8]. While most nuclear-powered countries are developing national GDFs, some are also exploring multinational storage solutions [4,8].

## 3. Geological Disposal Facilities

### 3.1. Overview

In 2021, the largest producers of nuclear energy were the United States, France, China, Russia, and South Korea. Additionally, countries such as Ukraine, Slovakia, Hungary, Czech Republic, Finland, Switzerland, and Belgium generated more than 25% of their electricity from nuclear power plants [17].

As of 2026, geological disposal of spent nuclear fuel has not yet commenced anywhere in the world. Finland’s ONKALO facility is approaching operation, while Sweden has begun construction of its spent-fuel repository at Forsmark and Canada has entered the regulatory decision-making stage for its selected repository site. In the meantime, HLW is predominantly stored in wet storage pools and dry cask storage systems, which are interim solutions designed to contain and shield radioactive materials [18].

The design and planning of a GDF involves a systematic and comprehensive process addressing technical, environmental, and regulatory aspects. Site selection requires extensive geological assessments, focusing on rock stability, low permeability, and minimal seismic activity to ensure long-term containment. Following site selection, the GDF is constructed using a multi-barrier system, which incorporates the following:

- Engineered barriers, including corrosion-resistant canisters (copper, steel, or titanium), buffer materials (e.g., bentonite clay), and cementitious backfills to limit water movement.
- Natural barriers, provided by the host rock, which acts as an additional containment layer by restricting radionuclide migration [5].

This multi-layered containment strategy enhances repository safety by providing redundancy in defence mechanisms, significantly reducing the likelihood of radioactive release and ensuring the long-term isolation of nuclear waste.

### 3.2. Planning

At present, over 30 countries with nuclear power plants are engaged in various stages of GDF implementation, including feasibility studies, site selection, geological characterisation, repository design, and licensing efforts [5]. However, only a few nations, such as Finland, Sweden, Canada, and France, have made significant progress towards establishing operational repositories.

During the planning stage of GDF development, careful consideration is given to site selection, feasibility assessments, and the integration of engineered and natural barriers. Globally, every proposed disposal concept incorporates a multiple-barrier system, which ensures redundancy in containment mechanisms to prevent the migration of nuclear waste [19].

The design and construction of a GDF is an intricate and highly regulated process, essential for the permanent isolation and long-term safety of HLW. Planning efforts must address environmental, safety, and regulatory requirements, ensuring that the repository meets stringent international standards. These processes involve a series of structured steps. Initial efforts focus on site selection and characterisation, involving extensive geological surveys and hydrogeological assessments to identify stable formations capable of long-term isolation. Vertical boreholes may also be used to obtain detailed mineralogical, geochemical, petrophysical, hydraulic, and geomechanical data [20].

#### Siting

The siting process for nuclear installations and GDFs follows several structured stages. The process begins with a site survey, during which broad regions with potentially suitable geology are identified. This is followed by site selection, where candidate sites are evaluated and ranked based on safety, geological stability, and feasibility studies. The site characterisation involves detailed hydrogeological, geomechanical, and geochemical assessments to confirm the site’s suitability [21,22]. This phase also establishes the site-specific design basis for repository construction. The pre-operational stage focuses on confirmatory studies and baseline environmental monitoring to ensure the site’s long-term geological stability. Once the repository is operational, continuous safety assessments and long-term monitoring are carried out to verify the integrity of the disposal system [21,22].

For GDFs, the selection of a suitable geological setting is critical for ensuring long-term containment. The host rock must provide stability, low permeability, and resilience against geodynamic processes such as earthquakes and glacial cycles [23]. Passive safety is largely dependent on the geological formation’s natural ability to act as a containment barrier, preventing the migration of radionuclides and enhancing the repository’s overall safety [23]. The role of geological barriers and their specific characteristics will be explored in greater detail in a later section.

### 3.3. Multi-Barrier Approach

**Figure 2:**
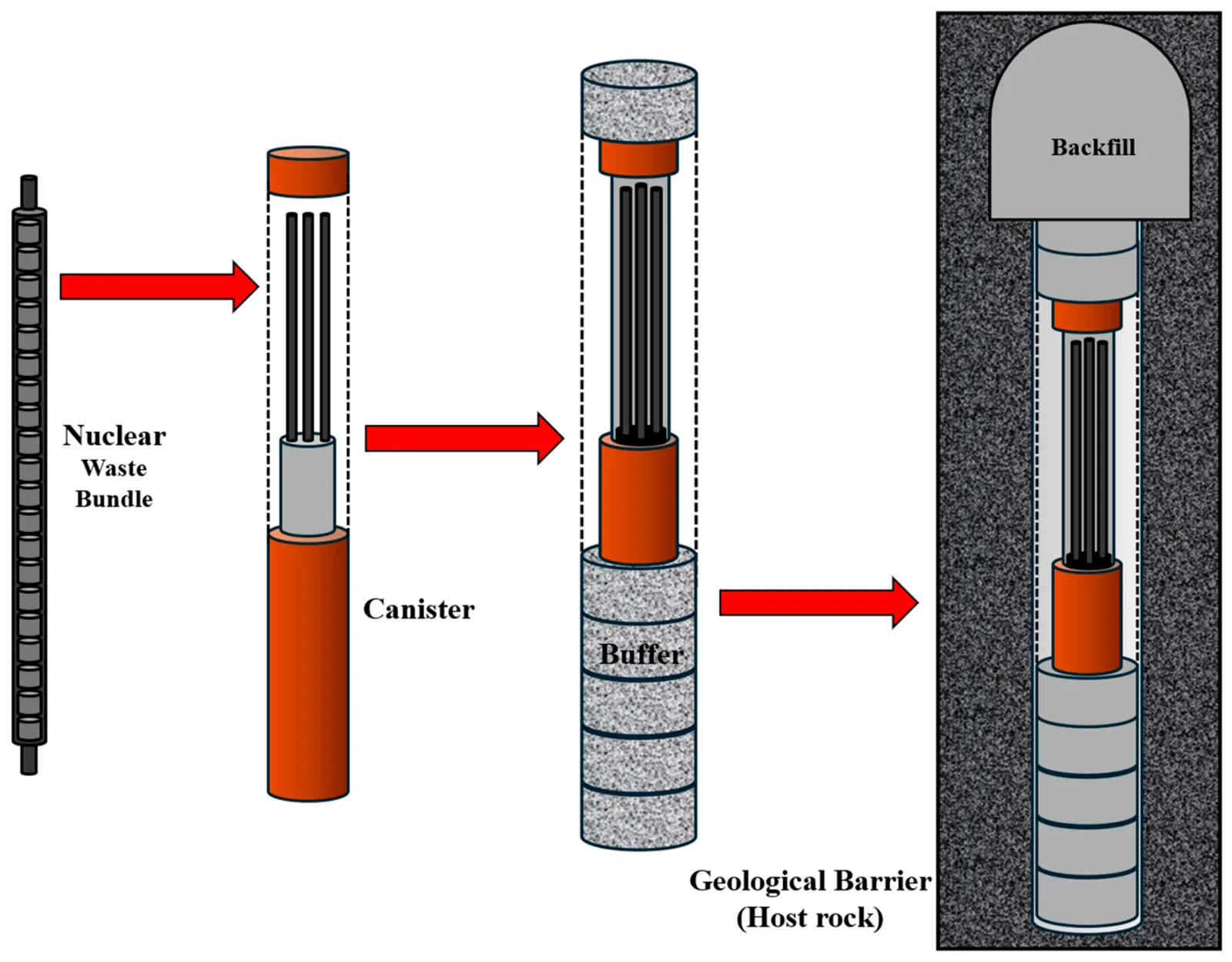
Schematic of the GDF multi-barrier system and assembly sequence, highlighting the radioactive waste form, metallic canister, buffer material, repository backfill, and surrounding geological host rock. Red arrows indicate the progressive emplacement of the waste package within the engineered and geological barriers.

The multi-barrier system is a fundamental concept in the design of GDFs for the safe disposal of nuclear waste. This system consists of a combination of engineered and natural barriers working in conjunction to mitigate the transport of radionuclides into the biosphere, ensuring long-term containment [5,9]. Multiple barriers provide redundancy so that if one safety function fails, additional mechanisms compensate to maintain overall security [4]. These barriers, depicted in Figure 2, commonly include the waste form, a metal canister encapsulating the waste (often copper or carbon steel with a cast-iron insert), a buffer material such as bentonite, a backfill material (often bentonite or cementitious fill), and the surrounding host rock. The selection of appropriate barriers is critical, as no single barrier should compromise the performance of another [24]. The repository is typically located at depths between 500 and 1000 m, although specific designs vary based on geological conditions and national regulations. As illustrated in Figure 2, the engineered barrier system therefore comprises the waste form, canister, surrounding buffer and repository backfill, while the host rock provides the principal natural geological barrier.

#### 3.3.1. Geological Barriers

The geological barrier plays a fundamental role in the multi-barrier containment system of a GDF. While the siting process ensures the selection of a stable and geochemically suitable host rock, the geological barrier itself serves as a long-term defence against radionuclide migration, complementing the engineered containment measures. The low permeability and sorption capacity of certain rock types, such as clay formations, restrict groundwater movement and provide an additional layer of protection. Crystalline rock formations, such as granite and gneiss, offer high mechanical stability, preventing structural disturbances that could compromise the integrity of the repository [25]. Salt formations, including rock salt and halite, also present favourable conditions due to their self-sealing properties, extremely low permeability, and plastic behaviour, which allows the rock to creep and close fractures over time.

Beyond acting as a physical barrier, the geological formation interacts with the engineered barriers to enhance containment. In clay-based GDFs, such as those planned in France and Switzerland, the host rock provides an additional diffusion-limited environment, slowing the transport of any radionuclides that may escape from the engineered barriers. In contrast, crystalline rock repositories, such as those in Sweden and Finland, rely more heavily on the buffer material (e.g., bentonite clay) to compensate for natural fractures in the rock. The geochemical properties of the host rock also influence long-term stability, as certain formations promote the precipitation of insoluble radionuclide compounds, further limiting mobility within the repository [25].

The geological barrier also plays a role in limiting microbial activity, which could otherwise influence repository conditions [7]. In clay-based formations, the limited pore space and low oxygen availability suppress microbial populations that contribute to metal corrosion. However, in crystalline rock environments, deep groundwater flow may introduce microbial communities, necessitating careful monitoring of their potential interactions with engineered barriers. Understanding these long-term processes is essential for ensuring the effectiveness of the geological barrier in maintaining the isolation of nuclear waste over timescales of hundreds of thousands to millions of years [25].

#### 3.3.2. Engineered Barriers

##### Canister

At the core of a GDF is the waste canister, which serves as the primary containment system for radioactive waste. Canisters must withstand mechanical stress, thermal loads, radiation exposure and potential chemical interactions while ensuring that hazardous materials remain securely confined for thousands of years.

Canister construction concepts have normally followed one of two approaches, single-material construction or dual-walled designs [26]. Sweden and Finland have historically developed variants of the KBS-3 concept based on a load-bearing insert surrounded by a 50 mm thick copper overpack; earlier designs incorporated a cast-iron inner vessel to provide structural integrity [27]. The Swedish design has since been updated, with SKB specifying a steel insert while retaining the copper outer shell [28]. Similarly, the Mark II, developed by Canada, is a dual-walled design incorporating copper-coated carbon steel. This design involves a pill-shaped container with hemispherical heads and a carbon-steel inside that provides structural stability for the outer copper coating [27]. The coating process, often applied through electrodeposition or cold spray techniques, ensures the steel remains isolated from any groundwater interactions [29].

While dual-walled canisters provide enhanced corrosion resistance, their complex fabrication introduces potential risks, such as weld defects, coating delamination and structural failure under repository conditions. These challenges necessitate further optimisation before industrial-scale deployment [26]. Several other countries, including Switzerland, France, Belgium and Japan, have also investigated dual-walled approaches. Belgium’s ‘supercontainer’ concept consists of a carbon steel overpack surrounded by compacted bentonite clay and reinforced concrete, creating a multi-layered containment system that enhances passive safety [29]. In this design, the carbon steel forms a protective oxide layer when exposed to the alkaline pore water from bentonite, further minimising corrosion risks [30].

##### Buffer and Backfill

Although the same material can be used for both buffer and backfill each serves different, but complementary, purposes. The buffer material, usually highly compacted bentonite, surrounds the canister to provide physical, chemical, hydrological, and biological isolation. Its primary function is to prevent radionuclide release, absorb mechanical stresses, and limit microbial activity, thereby safeguarding the integrity of waste containers [5,6]. The backfill, in contrast, fills void spaces within the repository to provide structural stability, thermal regulation, and long-term mechanical support. By enhancing thermal conductivity, backfill materials assist in dissipating the heat generated by decaying nuclear waste, preventing excessive temperature fluctuations that could affect repository stability [6].

Bentonite clay is widely recognised as the benchmark material for both buffer and backfill applications due to its exceptional mineralogical, physicochemical, and mechanical properties [7]. Its low permeability significantly reduces groundwater infiltration, while its high swelling capacity enables it to self-seal cracks and fractures, preventing radionuclide migration. Additionally, its ion exchange capacity enhances radionuclide retention, while its thermal and compaction properties contribute to the repository’s long-term performance [5,31]. However, it is important to note that the swelling behaviour of bentonite is affected by factors such as water chemistry, dry density, and long-term mechanical stress, all of which require careful evaluation when designing a repository system.

### 3.4. Environmental Conditions

Following closure, GDFs undergo a progressive evolution in temperature and oxygen levels over time. Initially, the substantial release of heat due to radioactive decay from HLW leads to a rise in temperature at the interface between the clay and canister, often exceeding 90 °C depending on repository depth and heat load [32,33]. This stage, known as the dry-out phase, is characterised by low thermal conductivity due to unsaturated bentonite, resulting in limited heat dissipation [34]. The environment remains aerobic during this phase, as oxygen is not yet consumed.

During the “transition phase,” the bentonite buffer begins to saturate and swell, increasing thermal conductivity and gradually reducing temperature. Concurrently, oxygen is consumed by processes such as metal corrosion and microbial activity, with depletion rates varying from several months to multiple years depending on host rock properties and microbial activity [19,35].

In the “saturated phase,” all oxygen is depleted, resulting in a fully anoxic environment. The bentonite buffer becomes fully hydrated, further enhancing thermal conductivity and stabilising temperature conditions inside the repository [34]. Additionally, any porewater inside the bentonite gradually reaches chemical equilibrium with the surrounding groundwater, influencing long-term geochemical conditions.

### 3.5. Global Development of GDFs

Significant progress has been made in the implementation of geological disposal programmes in recent years, although the stage of development varies considerably between countries. Finland and Sweden currently have the most advanced programmes for the geological disposal of spent nuclear fuel, while Canada, France, and the United Kingdom remain at different stages of licensing, site characterisation and repository development.

Finland is currently the most advanced country in the implementation of geological disposal for spent nuclear fuel. Posiva Oy is developing the ONKALO final disposal facility at Olkiluoto, which comprises an encapsulation plant and an underground repository located at a depth of more than 400 m. During 2025, installation and commissioning activities continued, alongside full-scale testing of the disposal process using non-radioactive fuel-element replicas. Production of components required for disposal, including canister components and bentonite-based tunnel backfill, has also commenced. Posiva aims to achieve readiness to begin final disposal during 2026, subject to completion of the operating-licence process [36].

Sweden has also progressed from repository planning to construction. The Swedish Nuclear Fuel and Waste Management Company (SKB) began construction-related activities for the Spent Fuel Repository at Forsmark in January 2025. The repository will be constructed at a depth of approximately 500 m in crystalline bedrock and is planned to accommodate approximately 12,000 tonnes of spent nuclear fuel in around 6000 copper canisters. Initial construction involves preparatory works above ground, followed by excavation of the underground repository. Disposal of spent nuclear fuel is expected to commence during the 2030s, with repository development continuing progressively for several decades [28,37].

Canada has also reached a major milestone in the development of its deep geological repository. In November 2024, the Nuclear Waste Management Organization (NWMO) selected the Revell site in northwestern Ontario, with Wabigoon Lake Ojibway Nation and the Township of Ignace as the host communities for Canada’s first deep geological repository for used nuclear fuel. Following site selection, the programme has progressed into the regulatory decision-making stage, with repository design and technical planning being adapted to the site-specific conditions. The project will require environmental and regulatory assessment and nuclear licensing before construction can begin [38].

The United Kingdom remains at an earlier stage of GDF implementation, with a final repository site yet to be selected. Nuclear Waste Services (NWS) continues to develop the geological disposal programme through site evaluation, design development, safety-case preparation and planning for future intrusive site investigations. Regulatory engagement between NWS, the Environment Agency and the Office for Nuclear Regulation is ongoing, with the regulators currently providing pre-application advice and preparing for future permit and licence applications. The first environmental permit application associated with intrusive site investigations is currently anticipated at approximately 2028, although significant programme and siting uncertainties remain [39].

France is developing the Cigéo deep geological repository for the disposal of high-level and intermediate-level long-lived radioactive waste. The repository is planned within a clay-rich geological formation at a depth of approximately 500 m. Andra submitted the application for authorisation to construct Cigéo in January 2023, and the French Authority for Nuclear Safety and Radiation Protection (ASNR) completed its technical review of the application during 2025. ASNR concluded that the preliminary safety case had reached a sufficient level of maturity for the current stage of the project, while identifying additional information and studies required during subsequent stages of repository development. The project therefore remains within the authorisation process prior to construction and eventual operation [40].

Collectively, these programmes demonstrate the substantial variation in GDF development worldwide. Finland is approaching the transition from construction and commissioning to disposal operations, while Sweden has entered the construction phase. Canada, France, and the United Kingdom remain within regulatory, licensing, or site-development stages. Continued progress in these programmes will provide important operational and technical evidence for the long-term performance of engineered barrier systems and candidate canister materials.

## 4. Corrosion Mechanisms Inside GDFs

Corrosion of metallic canister materials in GDFs is governed by chemical and electrochemical reactions that depend on factors including moisture, groundwater chemistry, redox conditions, and the electrochemical state of the metal [41,42]. Under repository conditions, both general and localised corrosion, together with environmentally assisted degradation processes such as hydrogen embrittlement and stress corrosion cracking, may influence long-term canister performance.

### 4.1. Abiotic Corrosion of Canister Materials

#### 4.1.1. Fundamentals of Corrosion

In the context of corrosion, anodic and cathodic half-reactions are fundamental components of the electrochemical processes governing the degradation of metals. The anodic half-reaction involves the oxidation of metal atoms, releasing electrons into the surrounding environment. The general anodic half reaction describing metal dissolution is shown below, *M*, in aqueous media, resulting in ion *M^z+^* (and the equivalent number of electrons *ze^−^*) [42].

(1) $$M\to M^{z+}+ze^{-}$$

On the other hand, the cathodic half-reaction entails the reduction of an electrochemically active oxidant, such as oxygen, leading to the formation of stable compounds [42]. A common cathodic half-reaction involving the reduction of oxygen is shown below.

(2) $$O_{2}+4e^{-}+4H^{+}\to 2H_{2}O$$

Overall, the complete corrosion reaction is the combination of the anodic and cathodic reactions.

Understanding corrosion is paramount for GDF applications, as it enables the development of effective mitigation strategies, ensuring the long-term integrity and safety of nuclear waste canisters in complex and challenging subsurface environments. Five principal forms of corrosion and environmentally assisted degradation relevant to GDF canisters are considered below.

- Uniform corrosion;
- Pitting corrosion;
- Crevice corrosion;
- Hydrogen embrittlement;
- Stress corrosion cracking.

These classifications highlight the different mechanisms through which corrosion can occur, each of which may pose specific challenges in environments such as that presented inside a GDF.

#### 4.1.2. Uniform Corrosion

**Figure 3:**
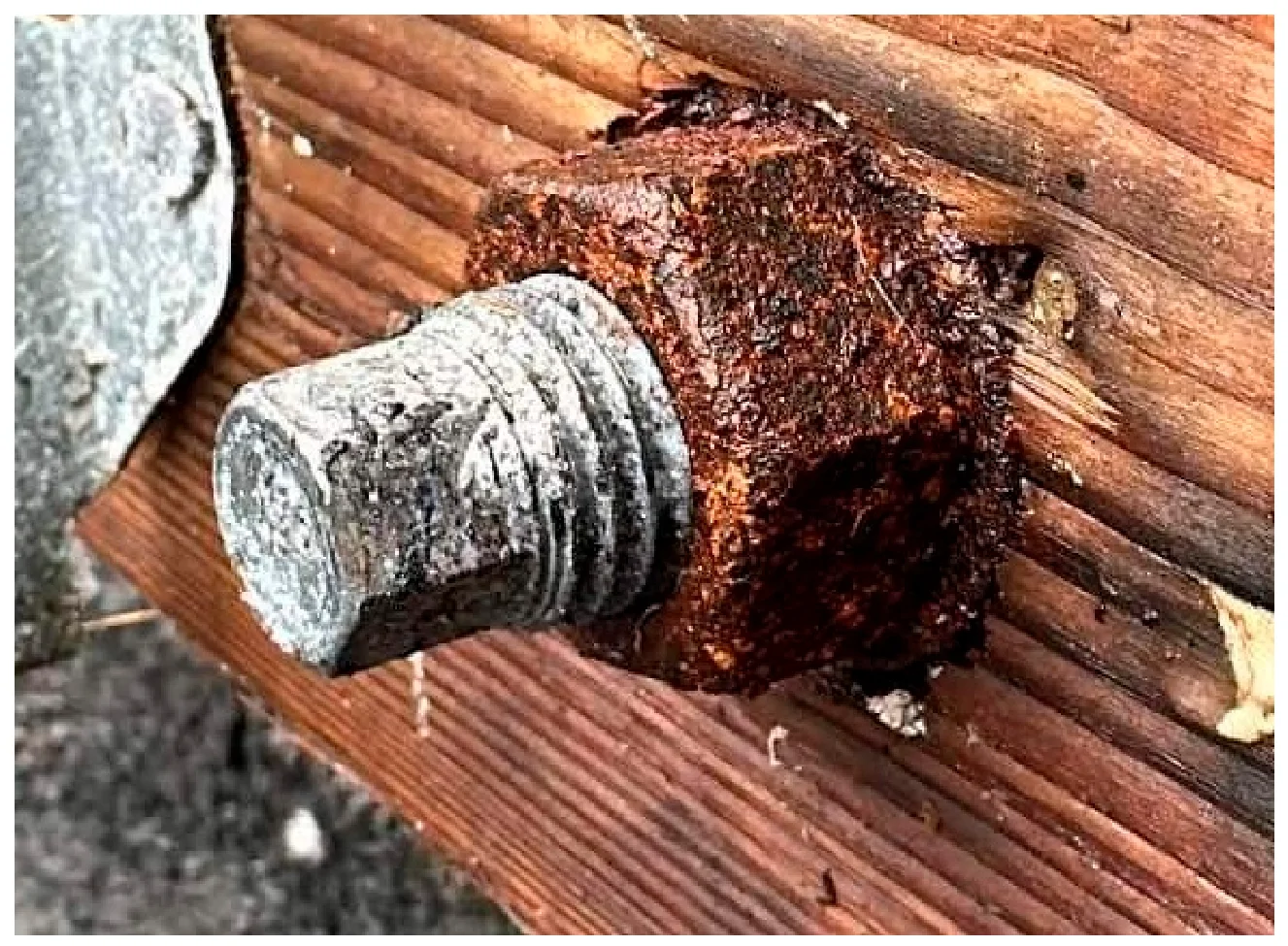
Example of uniform corrosion. Image depicts a corroded bolt. Photograph by the authors.

Uniform corrosion, also known as general corrosion, is a widespread degradation process that uniformly affects the entire exposed surface of a metal, leading to consistent material loss. This is recognised as the most common form of corrosion, representing the greatest destruction of metal on a tonnage basis [43]. Unlike localised forms of corrosion, such as pitting or crevice corrosion, uniform corrosion results in a steady and even deterioration of the metal across the entirety of its surface (see Figure 3).

The rate of uniform corrosion is influenced by multiple factors, including environmental conditions, moisture content, and the composition of the metal. For instance, metals in more aggressive environments, such as those with high chloride concentrations, may experience faster rates of uniform corrosion. While it can lead to a roughened appearance of the metallic surface, uniform corrosion is generally considered less damaging than other forms, as its impact on the life of equipment or structures can be accurately estimated through simple corrosion tests, such as weight loss measurements or electrochemical methods [44]. These predictive methods make uniform corrosion easier to monitor compared to localised forms of corrosion.

Although it is important to understand and carefully assess all types of corrosion for the design and successful implementation of a GDF, uniform corrosion is not of primary concern. This is predominantly due to the extensive knowledge of this process, which allows for relatively accurate predictions of corrosion rates over time, ensuring that the integrity of nuclear waste canisters can be effectively monitored and managed.

#### 4.1.3. Pitting Corrosion

**Figure 4:**
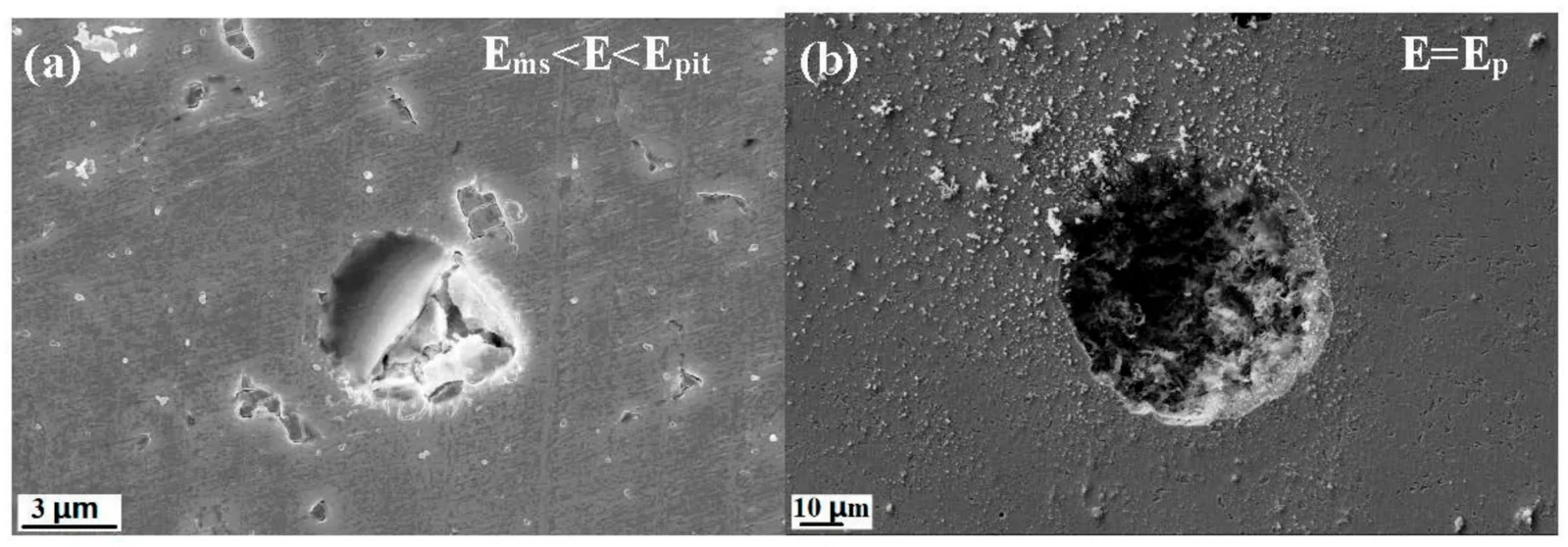
SEM images showing pitting initiation and development on X70 pipeline steel in a chloride-containing environment: (**a**) metastable pit formation below the pitting potential; and (**b**) developed pit morphology at the pitting potential. Adapted from Yang et al. [46] under the Creative Commons Attribution (CC BY 4.0) licence.

Pitting corrosion is a localised form of corrosion characterised by the formation of small, often microscopic, pits or craters on the metal surface (see Figure 4). Pitting represents a potentially severe form of localised attack that develops following breakdown of passivity and can penetrate deeply into the material [42]. These depressions result from the loss of passivity at specific sites, influenced by factors such as the existence of minority phases, halide, and hypochlorite ions [42].

Microbially influenced corrosion (MIC) can further accelerate pitting corrosion, as microorganisms can contribute to the breakdown of the protective oxide layer through the secretion of extracellular substances, such as organic acids, which facilitate localised corrosion [45]. Excluding MIC, pitting corrosion typically targets regions with surface abnormalities, such as scratches or welds, where the protective oxide layer may be compromised [45].

#### 4.1.4. Crevice Corrosion

Crevice corrosion is a localised form of corrosion similar to pitting and arises when the passivity of an oxide-covered metal is broken down within a confined area. A stagnant solution within a crevice can restrict oxygen transport to the metal surface, inhibiting repassivation of the protective oxide film [42]. Consequently, geometrical features such as welds, threaded components, metal-non-metal interfaces, and deposits of corrosion products or other solids can be particularly susceptible to crevice corrosion [45].

The restricted environment creates an electrochemical imbalance within the crevice, limiting the normal cathodic process described in Equation (2) and promoting localised metal dissolution [45]. The resulting accumulation of positive metal ions drives negative ions, including Cl^−^, from the bulk solution into the crevice. Hydrolysis of the dissolved metal ions subsequently generates *H*^+^ and lowers the local pH, further accelerating dissolution [45], as represented by the general reaction below:

(3) $$M^{z+}+H_{2}O\to MOH^{\left(z-1\right)}+H^{+}$$

The increase in hydrogen ions (*H*^+^) inside the crevice lowers the pH, which accelerates the dissolution of the metal, further promoting the corrosion process [45].

#### 4.1.5. Hydrogen Embrittlement

**Figure 5:**
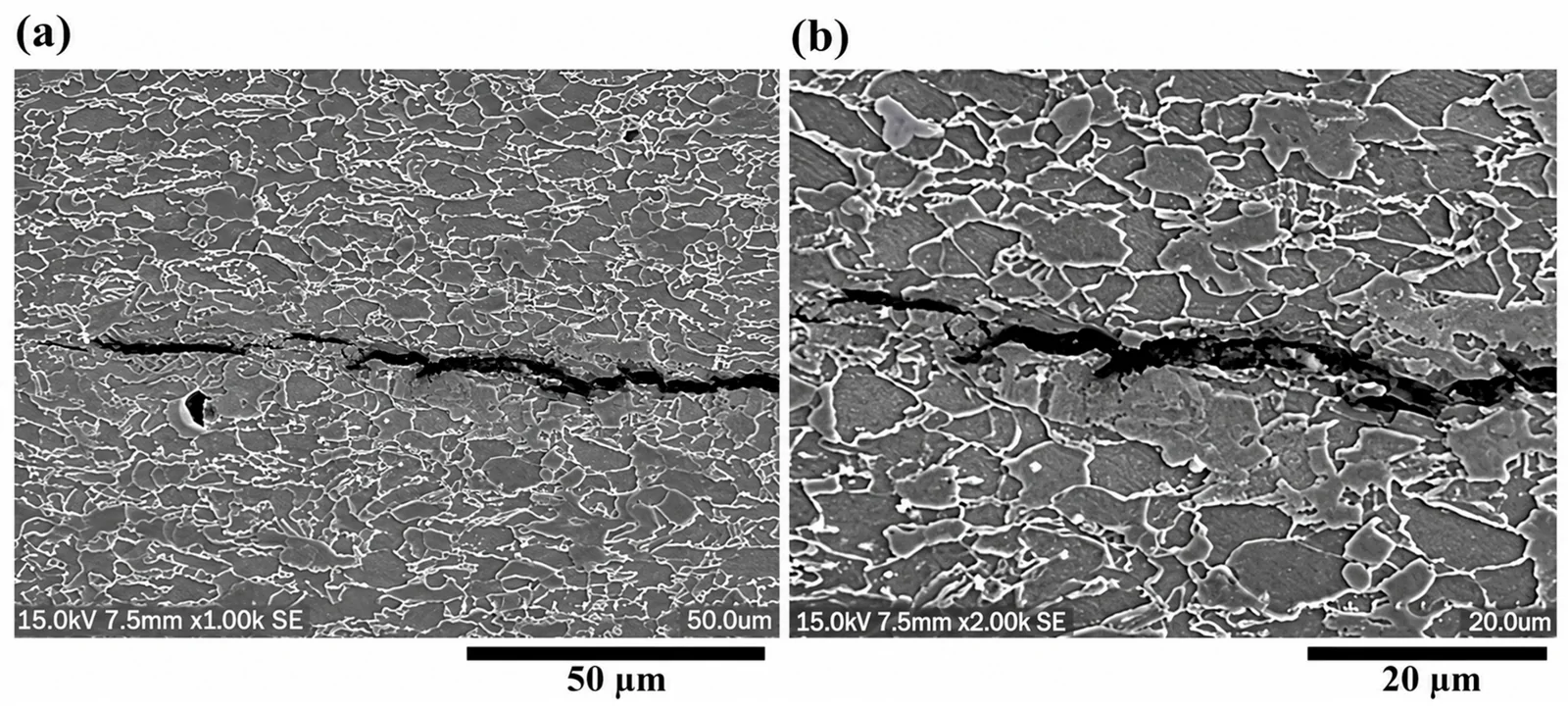
SEM images showing hydrogen-induced crack propagation in API 5L X70 pipeline steel: (**a**) overall crack morphology and (**b**) higher-magnification image of the crack path through the steel microstructure. Adapted from Mohtadi-Bonab et al. [49] under the Creative Commons Attribution (CC BY 4.0) licence.

Hydrogen embrittlement is a complex phenomenon affecting high-strength materials such as steel, titanium, and nickel-based alloys [42,47]. It occurs when hydrogen atoms infiltrate the metal lattice, making the material brittle and prone to cracking under stress (see Figure 5) [48].

Hydrogen can be absorbed into the metal during the fabrication of the component or as a result of the corrosion processes [48]. On the metal surface, hydrogen dissociates into atoms and enters the material through the sequential processes of adsorption and absorption. Adsorption occurs when hydrogen atoms adhere to the metal surface, as shown in the following reversible reaction [48]:

(4) $$H_{2}+2M↔2MH_{ads}$$

Once adsorbed on the metal surface, hydrogen can recombine into molecular *H*_2_ and either be released into the environment, or it can undergo a surface–subsurface absorption reaction and diffuse through the bulk material [48,50].

(5) $$MH_{ads}+MH_{ads}\to 2M+H_{2}$$

(6) $$MH_{ads}+M↔M+MH_{abs}$$

Once absorbed, hydrogen atoms can migrate between lattice sites and penetrate further into the material [48]. This can promote crack initiation and, under sufficiently severe conditions, contribute to structural failure [47].

Although the mechanisms of hydrogen adsorption, absorption, transport, and trapping are generally well understood, the physical mechanism causing hydrogen embrittlement is still a subject of scientific debate [47,48]. Some theories suggest that hydrogen causes embrittlement by accumulating at internal defects or grain boundaries, where it facilitates crack initiation. Stress-induced hydrogen cracking (SIHC) can then occur, exacerbating material failure under tensile stress [47,48].

#### 4.1.6. Stress Corrosion Cracking (SCC)

**Figure 6:**
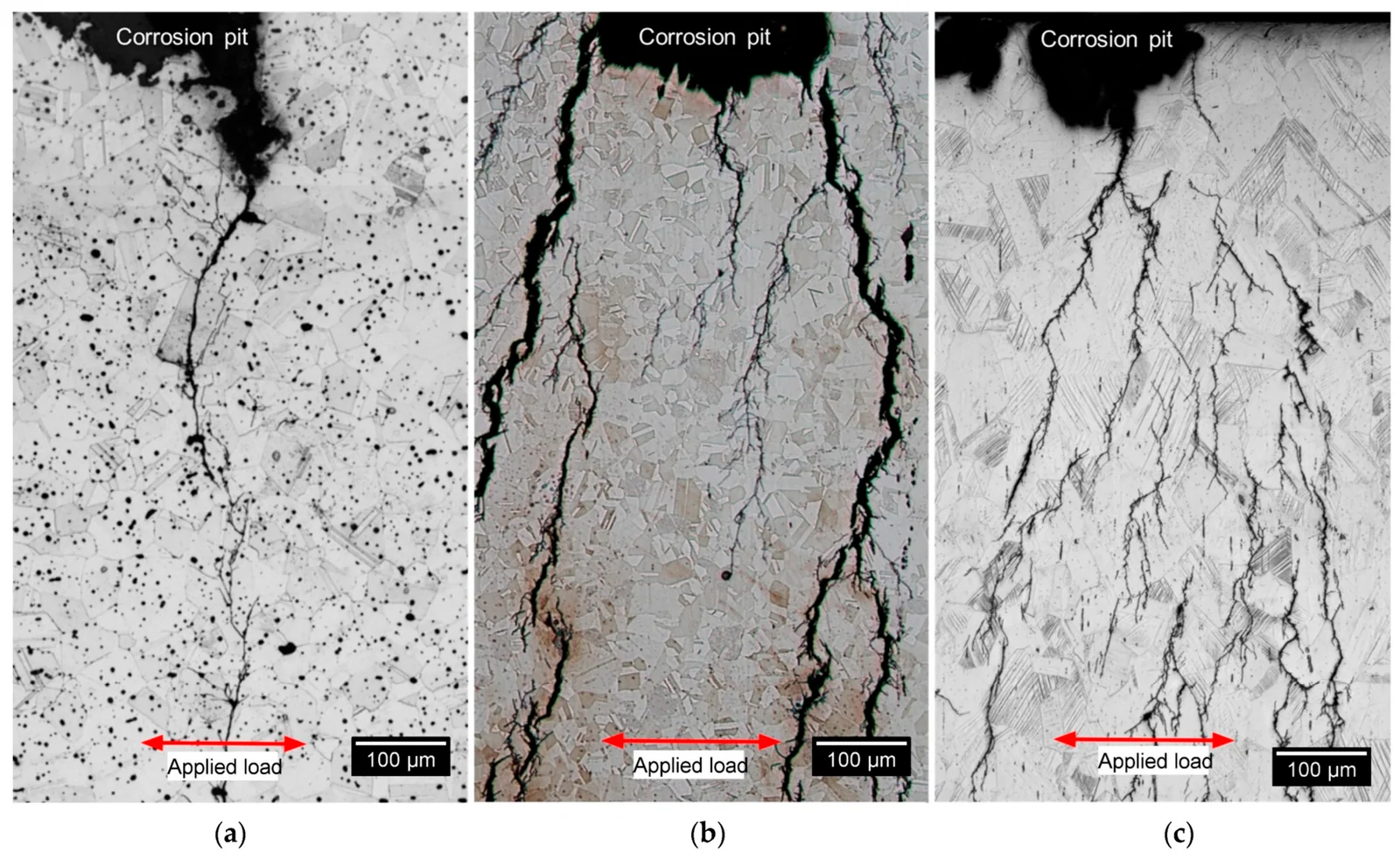
Micrographs showing stress corrosion cracking initiated from corrosion pits in 316L stainless steel under applied tensile loading. Panels (**a**–**c**) illustrate different crack morphologies propagating from localised corrosion sites. Adapted from Santamaria et al. [52] under the Creative Commons Attribution (CC BY 4.0) licence.

Stress corrosion cracking (SCC) is a phenomenon where the combined action of tensile stress and a corrosive environment leads to the spontaneous failure of metals at stress levels significantly lower than those required in non-corrosive environments (see Figure 6) [42,51]. For SCC to occur, three key conditions must be met: a susceptible material, a specific corrosive environment, and tensile stress. Under these conditions, cracks can form either inter-granularly (along the grain boundaries) or transgranularly (across the grains) [51]. These cracks result from the material’s exposure to a corrosive environment while under stress.

The initiation of SCC begins when a susceptible material is exposed to a corrosive environment, which causes microscopic cracks to form on the metal surface. As these cracks propagate under the influence of tensile stress, several mechanisms contribute to their growth. One such mechanism is stress-sorption, where the adsorption of corrosive species at the crack tip reduces the bond strength, facilitating further crack growth. Another key mechanism is film rupture-metal dissolution, where the protective oxide film on the metal surface is repeatedly ruptured and it cannot heal quickly enough, leading to localised dissolution of the metal and the extension of the cracks. Additionally, hydrogen embrittlement can occur, wherein hydrogen atoms infiltrate the metal, reducing its ductility and promoting brittle fracture [42,51,53].

As the cracks continue to grow, the material’s ability to withstand the applied stress diminishes, and eventually, failure occurs. This process can take years to manifest in real-world conditions, despite being able to develop rapidly under laboratory conditions. This emphasises the need for long-term monitoring and testing to detect SCC before it leads to catastrophic failure. Prevention of SCC involves controlling material susceptibility, environmental conditions, and applied stress. Strategies such as using resistant alloys, reducing the presence of corrosive agents, and minimising both residual and applied stresses can significantly reduce the likelihood of SCC occurring [42].

While these corrosion mechanisms can occur under abiotic conditions, microbial activity can further exacerbate them. MIC can accelerate uniform corrosion by altering local chemistry, promote pitting through biofilm-induced acidification, and increase susceptibility to environmentally assisted cracking through processes including hydrogen embrittlement. Understanding how MIC affects each candidate canister material is crucial for evaluating long-term repository safety.

### 4.2. Microbially Influenced Corrosion (MIC): Microbial Communities and Mechanisms

Microbially influenced corrosion (MIC) is defined as a type of corrosion that is facilitated or accelerated by the activities of microorganisms, such as bacteria, archaea, fungi, and algae. MIC is known to cause significantly higher corrosion rates than abiotic corrosion processes and is estimated to account for 20% of all corrosion damage, leading to annual costs of approximately USD 30–50 billion and contributing to 50% of all pipe infrastructure failures [54,55,56,57,58]. Bacteria can colonise surfaces, including metals and alloys which can lead to the formation of biofilms. These biofilms consist of immobilised cells encapsulated in an organic polymer matrix, containing solutes, heavy metals, inorganic particles, and other cellular constituents [59].

MIC poses a major threat to the integrity of high-level waste (HLW) canisters in GDFs [29]. Microbial corrosion can compromise the containment of radioactive materials, potentially resulting in the release of radionuclides into the environment, where they may become mobilised [7,19,54].

**Figure 7:**
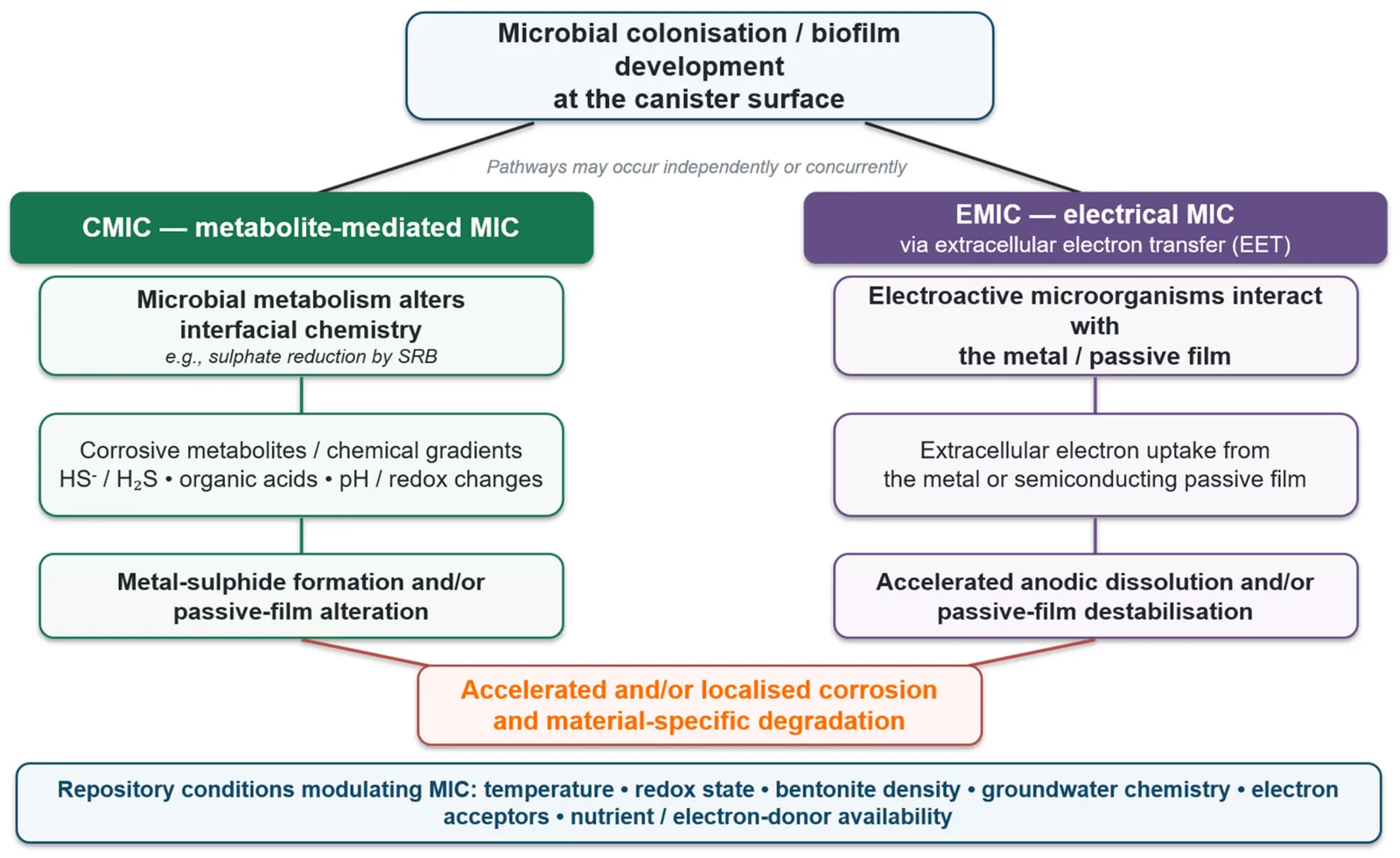
Conceptual overview of the principal CMIC and EMIC pathways relevant to metallic canister materials under GDF conditions, including key repository factors that may modulate MIC.

MIC can be characterised broadly into chemical microbially influenced corrosion (CMIC) and electrical microbially influenced corrosion (EMIC). CMIC involves indirect acceleration of corrosion through microbial metabolic products, including hydrogen sulphide generated by sulphate-reducing bacteria (SRB), or through microbial alteration of interfacial chemistry. In EMIC, electroactive microorganisms transfer electrons to or from extracellular solid substrates, including metal surfaces [59,60]. The key mechanism is extracellular electron transfer (EET), through which microorganisms can interact directly with the metal or passive film and alter its electrochemical behaviour [19,60,61]. The principal CMIC and EMIC pathways, together with the repository conditions that modulate their significance, are summarised in Figure 7.

Despite increasing research activity, knowledge of candidate canister performance under MIC in GDF environments remains limited [54]. Experimental conditions vary substantially among studies, including test media, microbial communities, nutrient availability, and exposure duration, which limits direct comparison. A more standardised approach to studying MIC under GDF-relevant conditions is therefore needed to support robust long-term safety assessment.

#### 4.2.1. Biofilm Formation

**Figure 8:**
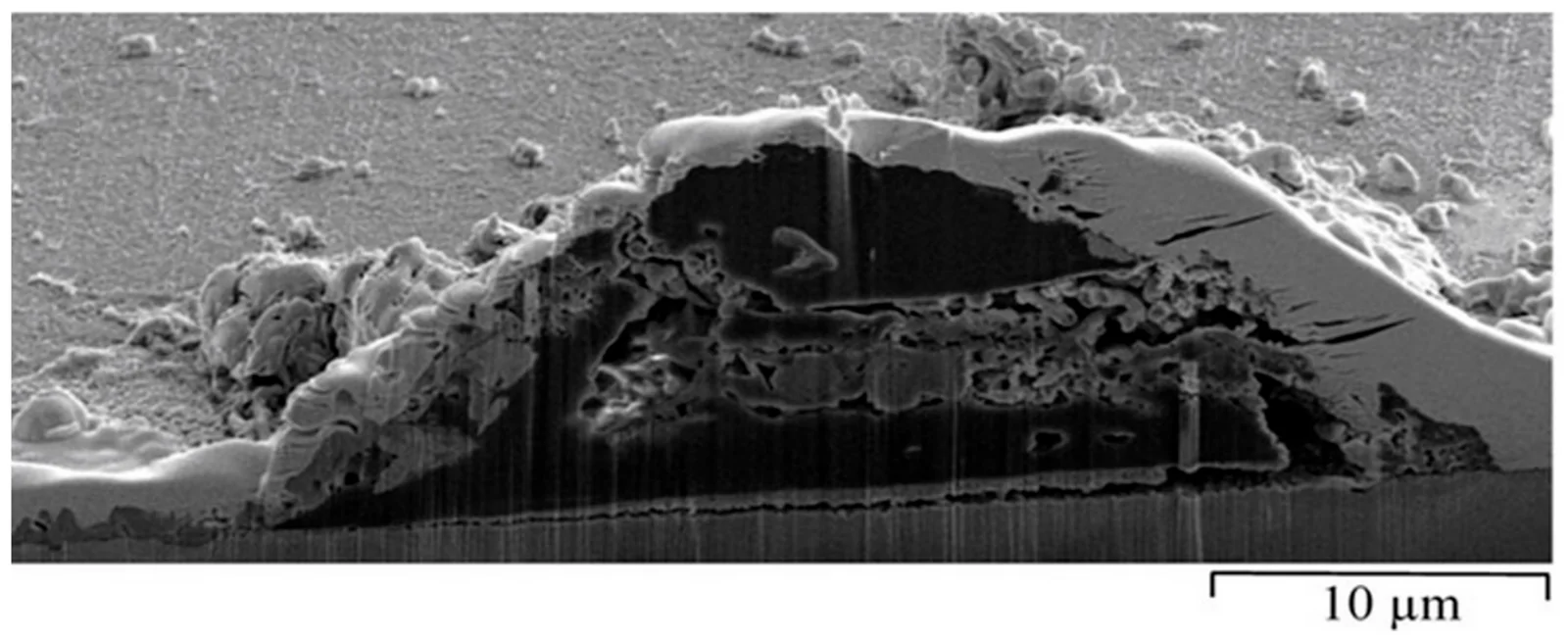
FIB-SEM cross-section of an SRB biofilm formed on carbon steel following 72 h exposure to a pure culture of sulphate-reducing bacteria in Postgate C medium. Adapted from Welikala et al. [65] under the Creative Commons Attribution (CC BY 4.0) licence.

Biofilm formation (see Figure 8) is a key process in MIC. Biofilms can protect microorganisms and establish local chemical environments that differ substantially from the surrounding bulk phase in pH, dissolved oxygen concentration, and organic or inorganic composition [59]. Microbial adhesion and growth are promoted by the production of extracellular polymeric substances (EPSs), which facilitate attachment to the metal surface and development of the biofilm matrix [7,56,62,63,64]. The establishment of significant microbial populations near waste canisters depends on factors including moisture, nutrient and energy availability, temperature, ionising radiation, and solute concentrations [55].

#### 4.2.2. Microbial Diversity

Microorganisms are highly adaptable and can colonise diverse environments, including the challenging conditions expected within GDFs. Although repository environments were previously assumed to become effectively sterile, evidence demonstrates that microorganisms can persist under radioactive, nutrient-limited, and deep subsurface conditions. In the aftermath of the Three Mile Island (TMI) nuclear power plant incident, water that covered the compromised reactor core revealed the presence of algae, fungi, yeasts, and bacteria [59,66]. This is further supported by the results of a study by Brown et al. (2015) [67] which highlights the existence of microbial activities under dose rates of gamma radiation similar to that emitted from radioactive waste. Additionally, a study by Petit et al. (2020) [68] detected the presence of 25 genera in the water supply of the radioactive core during operation, exhibiting activity levels exceeding 3 × 10^9^ Bq/m^3^. Lastly, microorganisms have been found to have the potential to endure such environments in a state of minimal metabolic activity or as spores [69].

Alongside this, the buffer material can also host microorganisms. For example, bentonite from the El Cortijo de Archidona region (Almería, Spain), which has been extensively studied due to its potential use in future Spanish GDF [70], has shown high microbial diversity [7,71,72,73,74]. Likewise, Opalinus Clay from Canton Jura, Switzerland (which has been considered in Swiss GDF design) revealed 5 × 10^6^ microbial cells per gram of clay, including SRB [75]. Furthermore, it has been reported that the organic matter inside bentonites has the potential to support microbial activity and processes [69].

Buffer materials and other barriers are not the only potential provider of microorganisms, as it has also been found that groundwater in GDFs often contains 10^1^–10^5^ colony-forming units/mL [59]. Construction of the GDF can also present the opportunity for the unintentional introduction of microorganisms. Together, these observations demonstrate that a GDF should not be assumed to be sterile. Consequently, understanding the microbial communities associated with repository barriers and their interactions with metallic surfaces is important for assessing MIC risk.

#### 4.2.3. Sulphate-Reducing Bacteria

After the closure of the repository, the transition to an anoxic environment starts with natural low levels of oxygen underground. This is further driven by the gradual consumption of oxygen within the GDF post-closure, driven by processes such as microbial activity and the corrosion of metal [7,69]. Eventually, an anoxic environment is reached in the repository. Under these conditions, some anaerobic bacteria can be active; for example, SRB, which are among the most commonly cited bacterial groups responsible for MIC and have the potential to induce pitting corrosion, which can be characterised as CMIC [54,76,77]. Some examples of SRB found in bentonite are *Desulfosporosinus*, *Desulfotomaculum*, *Desulfocapsa*, *Desulfuromonas*, and *Desulfovibrio* [74,78,79]. These bacteria use sulphate as the terminal electron acceptor (with the use of an electron donor, such as acetate or lactate) for respiration resulting in the formation and excretion of HS^−^ [80,81,82]. The overall reaction can be seen below [80]:

(7) $${SO}_{4}^{2-}+9H^{+}+8e^{-}\to HS^{-}+4H_{2}O$$

*HS*^−^ is a characteristic metabolite of SRB respiration and can either combine with *H*^+^ to make *H*_2_*S* or split into *H*^+^ and *S*^2−^, depending on pH [80].

(8) $${HS}^{-}+H^{+}⇌\;H_{2}S$$

*H*_2_*S* is highly corrosive and readily reacts with metallic surfaces to form metal sulphides. This reaction can lead to the degradation and pitting of the metal, and in the context of GDFs, it can result in the failure of the nuclear waste canisters. An example of this is the reaction of *H*_2_*S* with copper [80,82]:

(9) $$2Cu+H_{2}S\to {Cu}_{2}S+H_{2}$$

**Figure 9:**
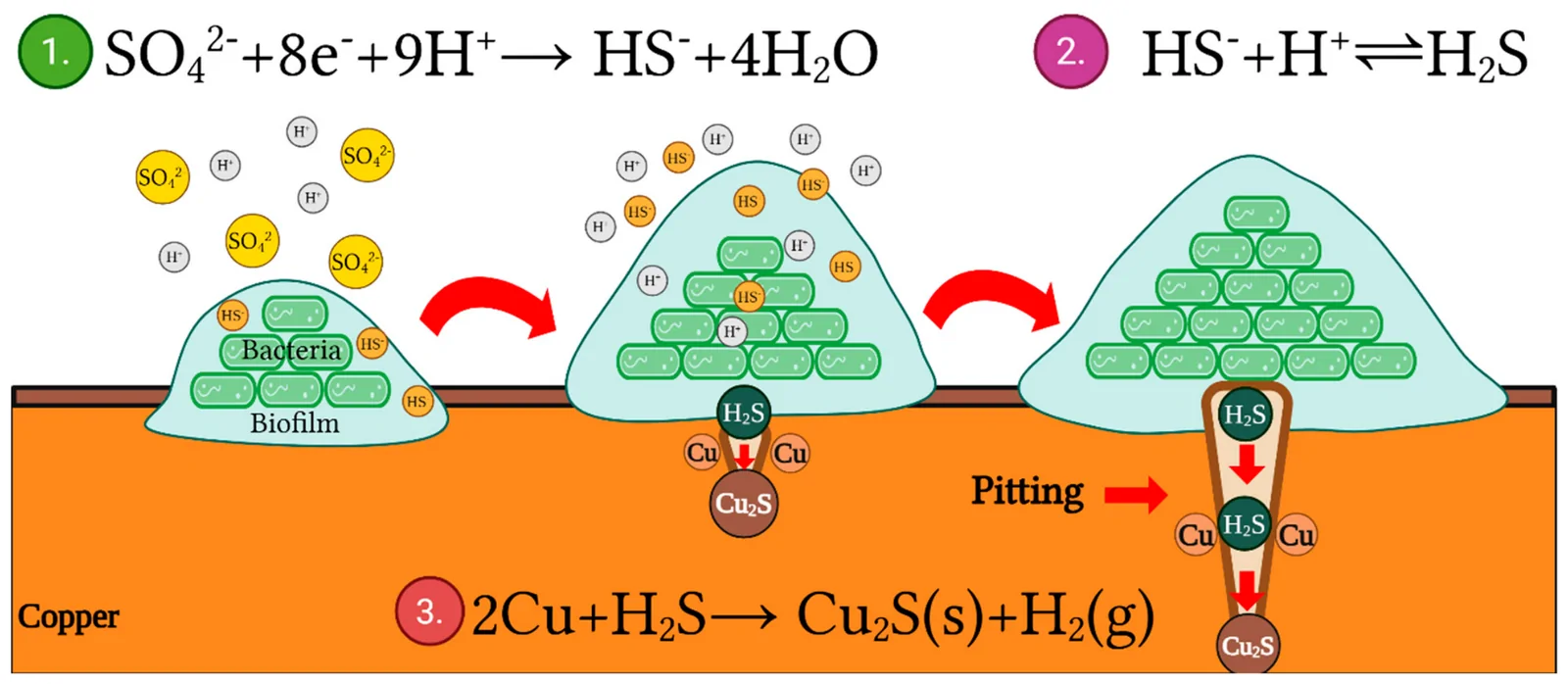
Visual example of the MIC of copper. Created in BioRender. Mumford, A. (2026) https://BioRender.com/oly0kfb.

This is also further demonstrated in Figure 9.

## 5. Candidate Canister Materials and Their Corrosion Mechanisms

The nuclear waste canister is an integral component of the engineered barrier system (EBS), providing a containment barrier that limits radionuclides migration into the biosphere [83]. Canister materials have typically been classified as either corrosion-allowance materials, such as carbon steel, or corrosion-resistant materials, including stainless steel, copper, titanium, and nickel-based alloys (see Table 2). Corrosion allowance materials typically exhibit uniform corrosion and demonstrate lower vulnerability to localised corrosion and environmentally assisted cracking [84]. In contrast, corrosion-resistant materials have a greater propensity for localised forms of deterioration, such as pitting or crevice corrosion [24]. Different materials are currently considered for canister construction; for example, in Sweden and Finland, copper is preferred due to its known corrosion resistance [6]. Carbon steel has been investigated as a strong option in countries such as France, Switzerland, Czech Republic, Slovakia, and Hungary [31,85]. Other materials include stainless steel, titanium, nickel, and ceramics [26,86].

To strengthen comparison between the principal canister materials considered in this review, Table 2 summarises representative grades, corrosion classification, principal degradation mechanisms, reported MIC pathways, representative performance evidence and the repository conditions most likely to control material behaviour. The table focuses on carbon steel, stainless steel, copper and titanium, while less-developed alternatives such as nickel-based alloys and ceramics are discussed separately in Section 5.5.

As shown in Table 2, no candidate material is universally resistant to degradation, with the dominant corrosion risk depending on both the material-specific protection mechanism and the evolving environmental and microbiological conditions within the GDF. Material selection must therefore consider mechanical robustness, corrosion resistance, radiation shielding, regulatory requirements, required containment lifetime, and compatibility with the surrounding engineered barriers [26,87]. In bentonite-based concepts, interactions between the canister material and buffer are particularly important because corrosion products and gas generation may influence the long-term performance of the wider barrier system.

### 5.1. Carbon Steel

The term carbon steel refers to alloys containing iron and carbon, with a carbon content below 2% by weight. Typically, this includes small amounts of manganese (below 1.65% by weight), silicon (below 0.60% by weight), and copper (below 0.60% by weight). Additionally, trace amounts of various alloying elements, including chromium, nickel, molybdenum, tungsten, vanadium, and zirconium, may be incorporated [26]. Carbon steel stands out as an appealing choice for canister material due to its multifaceted advantages, such as ease of fabrication, reliable corrosion performance, extensive industrial expertise, abundance, and relatively low cost [24].

Classified as a corrosion allowance material, carbon steel typically exhibits passivity when exposed to alkaline pore water. However, studies indicate that it can demonstrate active behaviour when in contact with compacted bentonite [6]. This dichotomy highlights the importance of a nuanced understanding of the material’s behaviour in diverse environmental conditions, such as GDF-relevant conditions. Multiple configurations for the use of carbon steel have been explored, with one notable approach involving its utilisation as a structural insert, complemented by an outer layer composed of materials like copper [6].

The corrosion characteristics of carbon steel offer a blend of advantages and obstacles to be considered when selecting a canister material. A principal advantage of carbon steel as a corrosion-allowance material is that uniform corrosion is expected to dominate under many abiotic repository conditions, enabling degradation to be treated relatively predictably in lifetime assessments [88]. Uniform corrosion can be accommodated through an appropriate wall-thickness allowance, whereas localised corrosion mechanisms are more difficult to predict and may produce disproportionately deep penetration [88]. A potential disadvantage is that carbon-steel corrosion products, including *H*_2_, Fe(II) species and expansive solid phases, may influence the properties of the surrounding buffer [88].

Repository conditions evolve substantially after closure, making it important to understand the corrosion of canister materials under aerobic and anaerobic conditions. Carbon steel will corrode aerobically and anaerobically, with the former being limited by the rate at which oxygen can move to the metal surface [88]. Under abiotic anaerobic conditions, the corrosion rate is limited by the rate of film growth on the surface of the material [88]. Repository conditions, however, cannot be assumed to remain abiotic.

Although carbon steel demonstrates a resistance to localised corrosion, this is not the case in biotic environments [59]. The most destructive culprit of MIC in most materials is SRB and this is no different for carbon steel. Four types of corrosion have been identified as potential concerns: pitting, SCC (induced by SRB), blistering, and hydrogen-induced cracking [59,89]. The pitting of carbon steel is stimulated by the production and release of *HS*^−^ by SRB. As mentioned in Section 4.2.3 Sulphate-Reducing Bacteria, *HS*^−^ can readily react with *H*^+^ to form *H*_2_*S*, which can react with Fe, resulting in corrosion product, iron sulphide, and hydrogen generation [90].

(10) $$Fe+H_{2}S\to FeS+H_{2}$$

Hydrogen generated during these reactions may also increase susceptibility to hydrogen embrittlement through enhanced hydrogen adsorption and uptake [26,59,88].

Although uniform corrosion is expected to dominate under many abiotic conditions, microbial activity can promote localised degradation. SRB are among the microbial groups most frequently associated with carbon-steel MIC and may contribute to pitting, hydrogen-induced cracking and other forms of localised attack [59,89]. Sulphide produced by SRB can react with iron to form iron sulphides while also generating hydrogen [90].

The MIC of carbon steel under GDF-relevant conditions has received increasing research attention in recent years. While some studies suggest carbon steel can remain passive in anoxic conditions, others highlight that microbial activity can significantly accelerate corrosion. Research has shown that specific microbial communities, indigenous to GDF environments, play a significant role in accelerating corrosion. Shrestha et al. (2021) [60] found that the presence of nitrate-reducing bacteria, particularly *Methyloversatilis*, led to significant localised corrosion of carbon steel in synthetic bentonite pore water, with localised penetration approaching 1 mm within 12 months. Similarly, Černoušek et al. (2019) [91] demonstrated that SRB, such as *Desulfomicrobium* and *Desulfovibrio*, accelerated corrosion through biofilm formation and the production of mackinawite (*FeS*), a corrosion product associated with the MIC of iron by SRB. Rajala et al. (2015) [92] observed that microbial communities in deep groundwater could derive energy from carbon steel, further contributing to corrosion, despite otherwise low rates in abiotic environments. In contrast, Diler et al. (2023) [93] showed that more alkaline cement-grout formulations in repository conditions reduced microbial biomass and diversity, resulting in lower corrosion rates, which suggests that environmental adjustments could mitigate MIC risks.

More recent studies have extended the focus of MIC beyond SRB to include other microbial groups, such as nitrate-reducing bacteria (NRB), which may also pose a significant risk to metal integrity in repository conditions. Shrestha et al. (2021) [60] demonstrated that even under anaerobic, nitrate-rich conditions representative of bentonite porewater environments, NRB—including genera such as *Methyloversatilis* and *Pseudomonas*—were capable of driving localised corrosion. Although this work focused on carbon steel, the findings underline the broader importance of NRB in MIC, particularly where nitrate or other oxidants are present. The study showed corrosion rates up to ten times higher than in abiotic controls, with localised attacks reaching depths of 1 mm within 12 months. These findings demonstrate that carbon-steel MIC cannot be considered solely in terms of SRB activity and that nitrate availability may alter both the dominant microbial community and the resulting corrosion mechanism.

**Table 2:** Comparative summary of principal candidate canister materials, representative grades, corrosion behaviour, and GDF-relevant microbiologically influenced corrosion (MIC) evidence.

Material/Representative Grades	Classification	Principal Corrosion Concerns	Representative MIC Organisms/Mechanisms	Representative Performance Evidence	Key GDF Controls	Programmes
**Carbon steel** P285NH overpack; API 5L X65 casing [93]	Corrosion allowance	Predominantly uniform corrosion under abiotic conditions; pitting, hydrogen embrittlement/HIC and possible SCC under aggressive conditions.	NRB including *Methyloversatilis* and *Pseudomonas*; SRB including *Desulfovibrio* and *Desulfomicrobium*. *FeS* formation, sulphide attack and *H*_2_ generation.	NRB exposure produced corrosion rates up to ~10× abiotic controls, with localised attack approaching 1 mm within 12 months [60]. More alkaline cementitious conditions reduced microbial biomass and corrosion [93].	pH/alkalinity; nitrate and sulphate availability; redox state; groundwater chemistry; microbial activity.	France, Switzerland, Czech Republic, Slovakia, and Hungary [31,85]
**Stainless steel** 304L (UNS S30403); 316L (UNS S31603) [9]	Corrosion resistant	Pitting, crevice corrosion and SCC following passive-film breakdown, particularly in chloride-bearing environments.	SRB and other biofilm-forming communities; sulphide generation and local acidification can destabilise passivity and promote localised attack.	Long-term buried stainless-steel studies detected microbial sulphate reduction with little measurable corrosion, whereas simulated repository studies showed biofilm-induced electrochemical changes and increased localised-corrosion susceptibility [94,95,96].	Cl^−^ concentration; temperature; sulphide; redox transitions; biofilm formation.	Belgium, UK [9]
**Copper** Oxygen-free phosphorus-doped copper (Cu-OFP); high-purity oxygen-free Cu based on UNS C10100 [97]	Corrosion resistant	Generally low uniform corrosion; sulphide-associated localised corrosion is the principal MIC concern.	Predominantly CMIC from SRB-generated *H*_2_*S*/*HS*^−^, producing *Cu*_2_*S*. Copper is comparatively resistant to direct electron-transfer mechanisms.	SRB biofilms produced corrosion rates up to 9.8 μm y^−1^ [98], whereas a 400-day bentonite study detected no significant MIC [99], illustrating strong environmental dependence.	Sulphide availability; temperature; bentonite density; nutrient/electron-donor availability; redox state.	Finland, Sweden; investigated in Japan and Switzerland [9]
**Titanium** ASTM Grade 2; Pd-containing Ti alloys [100,101]	Corrosion resistant/passive	Very low general corrosion; crevice corrosion for susceptible grades; possible localised passive-film disruption under reducing/sulphidic conditions.	*Desulfovibrio vulgaris*; sulphide-associated CMIC and possible electron-transfer effects under strongly reducing conditions.	Median abiotic corrosion rates of ~3–6 nm y^−1^, with reported values up to ~50 nm y^−1^ [9]. Grade 2 Ti exposed to *D. vulgaris* developed hemispherical pits up to ~2 mm in one study [101].	Sulphide concentration; pH/redox potential; temperature; biofilm development; passive-film stability.	UK, Germany, Japan, Belgium, Sweden, Canada [9]

**Note:** The table focuses on the four metallic material classes reviewed in depth in Section 5. Less-developed alternatives, including nickel-based alloys and ceramic concepts, are discussed separately in Section 5.5.

Despite advances in carbon-steel MIC research, extrapolation from accelerated laboratory experiments to repository performance remains challenging because bentonite compaction, groundwater chemistry, nutrient availability, and redox evolution can strongly constrain microbial activity. Field-scale evidence from the EURAD ConCorD programme illustrates this uncertainty: iron sulphides and elemental sulphur were detected after a seven-year steel exposure experiment at the Mont Terri Underground Research Laboratory, consistent with possible microbial involvement, whereas a separate in situ experiment using carbon-steel coupons embedded in MX-80 bentonite has, to date, shown no detectable microbial acceleration of corrosion [102]. More recently, Černá et al. (2026) demonstrated that bentonite-derived microbial consortia can substantially accelerate carbon-steel corrosion under nutrient-amended conditions, with nitrate-reducing systems producing localised attack; however, the authors emphasised that these experiments represented conservative, microbiologically favourable scenarios [103]. Collectively, these studies demonstrate that the presence of potentially corrosive microorganisms alone is insufficient to predict MIC severity and that repository-specific transport, nutrient, and buffer conditions must be considered.

### 5.2. Stainless-Steel

Stainless steel has been a contender for potential canister construction in various countries and programmes. Type 316L (UNS S31603) austenitic stainless steel was initially considered in the Belgian program, encased in Boom Clay (with a pore-water Cl^−^ concentration of 27 mg/L) [9]. However, this alloy was ultimately dismissed due to concerns about stress corrosion cracking (SCC) and localised corrosion induced by thiosulfate formation during exposure to aerobic conditions caused by the oxidation of pyrite in the host rock [9]. Notably, Types 304L (UNS S30403) and 316L have been explored as container materials for ILW in the U.K., as well as for the interim storage of HLW. Given the presumed substantial industrial expertise in the fabrication of large stainless-steel pressure vessels and other cylindrical components, the construction of canisters for the storage of HLW is anticipated to pose no significant challenges.

Despite their utility and ease of fabrication, stainless-steel containers face challenges, particularly their vulnerability to localised corrosion and SCC in the presence of chloride ions (Cl^−^), especially at elevated temperatures. The omnipresence of Cl^−^ in underground environments, coupled with the higher temperatures associated with heat-generating wastes, raises concerns about the potential for container failure. To mitigate this risk, the use of stainless-steel containers may necessitate the incorporation of a cementitious backfill. Notably, limited studies have been conducted on the behaviour of stainless steels under high-level waste/spent fuel (HLW/SF) disposal conditions, highlighting the need for further research in this critical area.

As already mentioned, microorganisms (specifically SRB) can be present under GDF-relevant conditions. Due to this, there is a real threat of MIC of any canister material selected for this application. Stainless steel can react similarly to carbon steel upon contact with *H*_2_*S*, resulting in corrosion products (see Equation (10)) [90].

Microorganisms, including SRB, may persist under GDF-relevant conditions and influence the passive surface of stainless steel. Sulphide generation and biofilm-associated changes in local chemistry can destabilise the protective oxide film and promote localised corrosion [55,90,104]. Consequently, the principal MIC concern is not sustained general corrosion, but local disruption of passivity under favourable microbial and geochemical conditions. Long-term studies on buried stainless steel have provided evidence of microbial activity in repository-relevant environments. Delwiche et al. (2007) [94] used radiographic imaging to confirm microbial sulphate reduction near stainless steel coupons buried for over 30 years, though measurable corrosion effects were minimal. Conversely, Horn et al. (2005) [95] demonstrated that biofilm formation in simulated repository conditions can significantly alter electrochemical properties, potentially creating sites of localised attack. Similarly, Féron and Crusset (2014) [96] found that MIC risk is most pronounced in transitional redox conditions, where microbial colonisation is enhanced by shifting from aerobic to anaerobic states. Once stable anoxic conditions are established, microbial activity may stabilise, potentially reducing MIC progression.

Recent evidence from nuclear-service environments further demonstrates that microbial effects on stainless steel are not uniformly detrimental. Shukla et al. (2025) investigated SS-304L exposed to bacteria isolated from spent nuclear fuel pool water and found that uniform biofilms formed by four bacterial isolates exhibited corrosion-inhibiting behaviour, whereas electrochemical measurements indicated that viable-but-non-culturable (VBNC) bacteria could contribute to localised corrosion [105]. This contrasting behaviour demonstrates that biofilm formation alone cannot be assumed to accelerate corrosion, with microbial physiological state and biofilm characteristics also influencing the response of the passive surface. However, spent nuclear fuel pool conditions differ substantially from the compacted, progressively anoxic environment expected around a GDF canister, limiting direct extrapolation to geological disposal. Taken together with repository-relevant studies [94,95,96], the available evidence suggests that the principal MIC concern for stainless steel is localised destabilisation of the passive film under favourable microbial and geochemical conditions, rather than sustained general corrosion. Long-term GDF-specific data nevertheless remain limited, particularly across the transition from aerobic to fully anoxic conditions.

### 5.3. Copper

Oxygen-free copper has been extensively investigated for GDF canister design since the 1970s and forms a central component of the Swedish and Finnish disposal concepts [9,27,106]. Copper has also been considered as a reference material in countries including Japan and Switzerland [9]. Its selection is supported by favourable corrosion behaviour under reducing repository conditions and by evidence from archaeological artefacts and naturally occurring metallic copper [9,27,107,108]. In recent years, the grades of copper deemed appropriate for canister design includes copper grades C10100-C15999, all containing minimum copper contents of ≥99.3% (*w*/*w*) [97]. Further adaptations of the alloy have been implemented over the past few decades, with oxygen-free copper with phosphorus (OFP) being developed to improve the creep rupture strain [9]. This is a non-standard grade, based on EN 1976:1998 [109] (equivalent to UNS C10100 [110]) but further doped with <5 ppm oxygen content (limited to improve weldability), 30–70 ppm phosphorus (to increase creep ductility), <0.6 ppm hydrogen (limited to ensure suitable ductility), and <8 ppm sulphur (limited to ensure suitable tensile strength and ductility) [97].

Studies suggest that copper can provide satisfactory containment, with estimates exceeding 100,000 years [26]. However, the suitability of copper is strongly dependent on repository redox conditions. Its favourable long-term behaviour is associated with the thermodynamic stability of metallic copper under sufficiently reducing, anoxic conditions, whereas persistently oxidising environments may sustain greater corrosion. Consequently, copper is less suited to repository concepts expected to remain oxidising, such as that proposed for Yucca Mountain [26]. On the other hand, it is compatible with saline conditions, due to the Cl^−^ ions promoting the active dissolution of Cu, suppressing the localised corrosion [9]. Therefore, it has been considered for repositories under the sea floor (e.g., Sweden and Finland).

In general, elemental copper and its compounds have shown a broad-spectrum of antimicrobial properties, by disrupting the plasma membrane or damage the DNA and proteins of Gram-negative and Gram-positive bacteria, fungi, and viruses [56]. Consequently, potential MIC of copper canisters for GDFs have received limited attention. Although copper is known for its bacterial resistance, it does not perform as adeptly with MIC, particularly when caused by SRB [80]. It has also been reported that copper is more susceptible to MIC than carbon steel [82]. The initial consideration of copper’s MIC resistance appears to be related to its resistance to EMIC, in which microorganisms are unable to extract electrons from the copper surface [111]. However, recent studies suggest that CMIC is most likely the reason for copper’s varied performance under MIC [112]. The mechanism of CMIC of copper by SRB begins with the excretion of *H*_2_*S*, which then reacts with copper [82]:

(11) $$2Cu+H_{2}S\to {Cu}_{2}S+H_{2}$$

This misconception of copper’s resistance to MIC grants continuation of studies into its performance in the presence of SRB. This is imperative to the integrity of any future GDF construction, particularly due to the overall present preference of this material for canister construction. Although some studies have appeared over the last few years [69,113,114], more of this nature must also be conducted under GDF-relevant conditions to ensure the safety of any GDF in the future.

Copper’s corrosion resistance makes it a strong candidate for GDFs, particularly in anoxic conditions. However, microbial activity, such as sulphide production, may still influence its long-term stability, requiring further investigation. The persistence of SRB in repository environments is a key factor influencing MIC risk.

Previous studies found that SRB could survive at 30 °C in compacted bentonite (1.7 g cm^−3^) but not at 60 °C, suggesting that temperature acts as a natural limit to MIC in some repository designs [69,113]. Microbial consortia enriched with sulphate and organic electron donors, such as acetate and lactate, were shown to produce sulphide, leading to *Cu*_2_*S* formation on copper surfaces after 1 year of incubation. Abramova et al. (2024) [98] further demonstrated that SRB biofilms actively corrode copper at rates of up to 9.8 µm year^−1^, showing that biofilm formation not only shields microbial cells from copper’s antimicrobial effects but also creates localised conditions that enhance corrosion. Key SRB families involved in this process include *Desulfomicrobiaceae*, *Desulfovibrionaceae*, and *Desulfuromonadaceae*, all of which are known to thrive under the reducing conditions expected in GDFs.

Despite these findings, the extent of MIC remains uncertain, as some studies have found minimal microbial influence on copper corrosion. Sushko et al. (2024) [99] reported no significant MIC effects after 400 days in static bentonite experiments, attributing any observed copper dissolution to abiotic geochemical interactions rather than microbial activity. These contrasting results suggest that MIC risk is highly dependent on site-specific conditions, including nutrient availability, electron donor limitations, and the physical properties of the bentonite buffer. While bentonite acts as a protective barrier by limiting microbial mobility, it can also serve as a microbial habitat if conditions allow for SRB proliferation. Tully et al. (2023) [115] demonstrated that increasing dry bentonite density from 1100 to 1600 kg m^−3^ reduced the initial corrosion rate of embedded copper, with corrosion rates decreasing to below 1 µm year^−1^ at longer exposure times. Importantly, although *Cu*_2_*S* was detected, the authors could not establish whether the sulphide source was microbiological or inorganic, demonstrating the difficulty of attributing sulphide-associated copper corrosion specifically to MIC without supporting microbiological evidence. Similarly, Werme et al. (1992) [116] demonstrated that even low concentrations of sulphide present in groundwater may contribute to copper corrosion over long timescales. This further highlights an important distinction in repository assessments: detection of copper sulphide corrosion products alone does not demonstrate MIC, because sulphide may originate from either microbial sulphate reduction or abiotic geochemical sources.

Martinez-Moreno et al. (2025) [117] investigated selenium interactions in bentonite and found that Se(IV), a long-lived fission product in HLW, inhibited SRB activity. As SRB drive MIC of copper through sulphate reduction, Se(IV) could suppress sulphide production, altering corrosion dynamics. However, some SRB can reduce Se(IV) to Se^0^, suggesting competition between selenium and sulphur for microbial reduction. This could limit sulphide availability, potentially mitigating MIC on copper while also restricting selenium migration in a GDF.

The long-term evolution of microbial communities in GDF environments remains one of the largest uncertainties in MIC risk assessments. While some studies suggest that microbial activity will remain low due to the harsh environmental conditions within the buffer, others indicate that SRB biofilms are highly adaptable and could persist for extended periods. Groundwater infiltration, redox fluctuations, and microbial succession over centuries or millennia could create conditions favourable for MIC, even if initial repository environments appear stable. These uncertainties highlight the need for further in situ studies that integrate microbiological, electrochemical, and geochemical analyses. Understanding how biofilms form, how microbial sulphide production progresses over time, and how trace geochemical variations influence MIC will be crucial for ensuring copper’s long-term viability as a canister material for nuclear waste disposal.

### 5.4. Titanium

Titanium has considered as a candidate canister material in several national programmes, including those in the U.K., Germany, Japan, Belgium, Sweden, and Canada [9]. A key consideration is the trade-off between the enhanced corrosion resistance of Pd-containing alloys and their higher cost [100]. Higher grade titanium alloys provide greater resistance to crevice corrosion, although lower grades may deliver satisfactory performance under less aggressive repository conditions [9,100]. Furthermore, titanium alloys have been deemed suitable for use with bentonite backfill or no backfill [9]. Titanium, being highly reactive and having a significant affinity for oxygen, forms beneficial surface oxide films spontaneously when fresh metal surfaces are exposed to air or moisture [100].

The outstanding corrosion resistance exhibited by titanium alloys is dictated by the creation of stable, continuous, highly adherent, and protective oxide films on the metal surface [100]. The instantly generated films, typically less than 10 nm in thickness and imperceptible to the naked eye, demonstrate remarkable chemical resistance, being susceptible to attack only by a limited set of substances [100]. The characteristics, composition, and thickness of these oxides are contingent on environmental conditions, with titanium dioxide (*TiO*_2_) being the predominant form in aqueous environments [100]. The corrosion behaviour of titanium alloys is predominantly dictated by the properties of *TiO*_2_ in the passive film [9].

Anhydrous conditions, in the absence of a source of oxygen, may lead to titanium corrosion, as the protective film may not regenerate if damaged. The study of titanium corrosion resistance essentially revolves around understanding the characteristics of the oxide film, which can instantaneously repassivate itself when traces of oxygen or moisture are present [100].

Titanium exhibits the widest range of passivity, with the stability of *TiO*_2_ influenced by redox potential and pH [9]. The corrosion rate remains statistically independent of temperature and solution composition within the studied ranges, with a median corrosion rate of 3 nm year^−1^ to 6 nm year^−1^, albeit with some values as high as 50 nm year^−1^ [9]. Failure of the container by general corrosion is unlikely except at very long times [9]. Pitting potentials of titanium alloys are significantly positive, yet susceptibility to crevice corrosion exists, especially for certain grades [9]. The commercially pure Grades 1 and 2 are clearly susceptible to crevice corrosion over the entire range of Cl^−^ concentrations at 95 °C [9]. The addition of palladium suggests a 20% increase in immunity to crevice corrosion up to at least 20% NaCl [9].

Despite titanium’s highly protective *TiO*_2_ passive film, two distinct microbial corrosion pathways should be separated. Unsal et al. (2023) provided experimental evidence for extracellular electron-transfer MIC (EET-MIC) by *Desulfovibrio vulgaris* under organic-carbon starvation [118]. In contrast, sulphide can also affect titanium through purely chemical interactions with the passive film. Yang et al. (2018) investigated this abiotic pathway in sulphide-containing simulated seawater and demonstrated incorporation of sulphur into the passive film as *TiOS* and *TiS*_2_ [119]. Representative reactions describing interaction of sulphide with the titanium oxide film are shown below:

(12) $${TiO}_{2}+{2HS}^{-}+{2H}^{+}↔{TiS}_{2}+{2H}_{2}O$$

(13) $${TiO}_{2}+{HS}^{-}+H^{+}↔TiOS+H_{2}O$$

Additional titanium-sulphide formation reactions considered in the same abiotic study are shown below [119]:

(14) $$Ti^{4+}+2S^{2-}↔{TiS}_{2}$$

(15) $$Ti^{4+}+S^{2-}+H_{2}O↔TiOS+2H^{+}$$

Importantly, Yang et al. [119] did not investigate microorganisms; therefore, these reactions should not themselves be interpreted as evidence of EMIC. In a GDF, however, equivalent sulphide chemistry could contribute to CMIC where sulphide is generated biologically by SRB. The effect of sulphide is concentration dependent, with low sulphide concentrations reported to support passive-film stability, whereas increasing sulphide promoted greater formation of sulphur-containing titanium species and reduced corrosion resistance [119].

Titanium’s strong passive oxide layer provides excellent corrosion resistance, making it a promising canister material. However, microbial activity could still influence its stability, particularly through biofilm formation or localised chemical changes, warranting further investigation. Historically, the predominant focus of titanium corrosion research has been on applications in areas such as aerospace, medical implants, and chemical processing. However, none of these applications precisely replicate the conditions encountered within a GDF. The long-term behaviour of the passive oxide film, under repository-specific conditions, needs to be further evaluated [9].

Titanium has historically been considered highly resistant to MIC because its stable *TiO*_2_ passive film limits interactions between microorganisms and the underlying metal [26,88]. However, experimental evidence indicates that this resistance is conditional rather than absolute. Rao et al. (2005) observed extensive colonisation and hemispherical pits of approximately 2 mm on ASTM Grade 2 titanium exposed to *D. vulgaris*, whereas Unsal et al. (2023) subsequently demonstrated EET-MIC under organic-carbon starvation [101,118]. These findings indicate that both sulphide-mediated CMIC and direct microbial electron-transfer mechanisms may contribute to titanium degradation under sufficiently favourable conditions.

More recently, Li et al. (2026) provided direct mechanistic evidence that the electroactive bacterium *Geobacter sulfurreducens* can reduce Ti(IV) within the *TiO*_2_ passive film, increasing film defect density and electronic conductivity and facilitating electron transfer from the underlying titanium [120]. This provides a mechanistic basis for EET-driven titanium MIC that is distinct from sulphide-mediated CMIC. However, these microbiological experiments do not reproduce the complete thermal, geochemical, and highly compacted bentonite environment expected in a GDF, limiting direct extrapolation to repository performance.

Evidence from other environments nevertheless demonstrates that microbial colonisation of titanium is feasible. Zhai et al. (2022) identified developed microbial communities on titanium exposed at a depth of 5772 m in the Yap Trench [121], although this does not in itself demonstrate significant GDF-relevant MIC. Separately, repository-focused work by Zhang et al. (2020) assessed hydrogen-related degradation of titanium under bentonite conditions [122]. Collectively, these studies emphasise the importance of distinguishing abiotic sulphide attack, sulphide-mediated CMIC, EET-MIC, and other degradation mechanisms when assessing titanium canister performance. The primary knowledge gaps that remain include the long-term stability of the passive film, as titanium repassivates rapidly in the presence of oxygen, but it is unclear whether biofilms could disrupt this process in long-term anoxic conditions. Additionally, the extent to which *TiS*_2_ and *TiOS* formation enhances or weakens titanium’s corrosion resistance is still debated. Over geological timescales, microbial populations in a GDF may adapt, potentially altering MIC risks in ways not yet fully understood [123]. Given these uncertainties, further long-term experimental studies are needed under GDF-relevant conditions to conclusively determine whether MIC could pose a risk to titanium over geological timescales. Future work should focus on in situ testing in GDF environments, investigating biofilm interactions under anoxic conditions, and assessing the evolution of microbial communities over extended timeframes.

### 5.5. Other Materials

Nickel alloys have been considered under a number of different programs, including Germany, Belgium, and the United States [88]. Ni-Cr-Mo alloys present the potential for exceptionally durable canisters, particularly when opting for an alloy that is either immune or highly resistant to localised corrosion within the repository environment [26].

Metallic materials are known for hydrogen generation and therefore their impact on other barriers inside the system [31]. Because of this, novel materials such as ceramics have been studied and considered for the application of canister construction. Despite the evident benefits of ceramics’ stability in various aqueous environments and their lack of hydrogen generation, there has been limited exploration of ceramics as a viable material for canisters [26]. The major issue involved with ceramic canisters lies in the difficulty of fabrication coupled with the material’s low fracture toughness [26].

In conclusion, as these metals are exposed to harsh and varied geological conditions, including the potential influence of microbial activity, characterising all of their corrosion processes is essential to predict their durability and performance. Although abiotic corrosion of these materials is very well documented and understood, there is a clear lack of studies of MIC of these metals under GDF-relevant conditions.

## 6. Future Research Recommendations for Canister Materials in GDF Environments Under MIC Conditions

Following the detailed assessment of candidate canister materials, it is clear that significant uncertainties remain in understanding their long-term performance in geological disposal facility (GDF) environments, particularly under the influence of microbiologically influenced corrosion (MIC). The interactions between engineered barriers, microbial communities, and site-specific environmental factors are inherently complex and dynamic, highlighting the need for targeted, interdisciplinary research.

While laboratory experiments provide valuable mechanistic insights, they often fail to replicate the evolving geochemical and microbial conditions present within a GDF. There is a pressing need for extended in situ studies in underground research laboratories such as Mont Terri, Grimsel (Switzerland), and the Äspö Hard Rock Laboratory (Sweden). These facilities provide a controlled yet realistic environment to study corrosion processes over timescales spanning years to decades. Emerging technologies—such as in situ electrochemical sensors, fibre optic probes, and coupled geophysical monitoring systems—may offer real-time insights into corrosion rates, redox dynamics, and microbial activity at metal interfaces. These tools have the potential to yield representative datasets for model calibration and improve the accuracy of long-term safety predictions.

The current diversity in MIC study methodologies—ranging from microbial strains and nutrient conditions to porewater composition and exposure duration—limits comparability and hinders cross-study evaluation. Standardising experimental designs using repository-specific conditions, representative microbial consortia, and defined exposure protocols would enhance reproducibility and facilitate benchmarking. Establishing common frameworks, supported by machine-readable data formats, would also enable broader data integration and support international modelling efforts.

In terms of predictive modelling, MIC is still underrepresented in safety assessment models for waste repositories. Incorporating microbial dynamics—such as biofilm growth, metabolic pathways (e.g., sulphate, nitrate, or iron reduction), and interactions with redox conditions—into mechanistic models is essential. In parallel, machine learning and AI tools trained on corrosion and microbial datasets could help identify behavioural trends and forecast long-term degradation under diverse environmental scenarios.

A major challenge remains the extrapolation of short-term laboratory data to repository timescales spanning thousands to millions of years. Addressing this will require hybrid approaches combining accelerated testing under repository-relevant stressors (e.g., elevated pressure, temperature, or radiation), natural analogue investigations, and in situ data. These strategies, coupled with mechanistic modelling, can improve confidence in long-term predictions.

Understanding the feedback mechanisms between microbial activity, corrosion products, and evolving environmental conditions is also critical. For instance, hydrogen gas generated by anaerobic corrosion can fuel hydrogenotrophic microbial metabolism, further exacerbating MIC. Similarly, microbial processes may alter pH, redox potential, and sulphide concentrations—factors that directly affect corrosion rates and surface stability. Future studies should aim to quantify these interactions across both engineered and natural barriers to inform system-level models.

Moreover, different canister materials exhibit distinct responses to microbial activity, underscoring the need for material-specific studies. Carbon steel is particularly prone to sulphide-induced pitting, local acidification, and hydrogen embrittlement. Stainless steel, though generally corrosion-resistant, may suffer from localised attack or stress corrosion cracking under MIC-associated redox shifts and in chloride-rich environments. Copper, while stable under anoxic conditions, is susceptible to sulphide-induced pitting when exposed to sulphide-generating microbes. Titanium, despite its highly stable passive oxide layer, may still undergo MIC-induced depassivation in strongly reducing environments or under dense biofilms. These degradation pathways are further influenced by microbial ecology, groundwater chemistry, bentonite interactions, and geochemical gradients.

To address these challenges, future research should prioritise the development of integrated experimental platforms that simulate coupled biogeochemical conditions and allow long-term monitoring of canister surfaces. These efforts are essential for refining predictive models, informing material selection, and supporting robust safety cases for the deep geological disposal of high-level nuclear waste.

## 7. Conclusions

The long-term performance of nuclear waste canisters cannot be assessed from material corrosion resistance alone, as degradation is governed by interactions between the canister material, evolving repository conditions, engineered barriers, and microbial activity. The evidence reviewed here demonstrates that no candidate material is universally resistant to degradation and that the dominant corrosion risk differs substantially between carbon steel, stainless steel, copper, and titanium. Importantly, the presence of microorganisms alone does not indicate that significant MIC will occur. Temperature, redox conditions, groundwater chemistry, bentonite density, nutrient and electron-donor availability, and the stability of corrosion products or passive films collectively determine whether microbial activity can influence canister degradation.

Carbon steel offers advantages in cost, fabrication, and the relative predictability of uniform abiotic corrosion, but microbial activity can shift degradation towards localised attack, particularly through sulphide production, nitrate reduction, and associated hydrogen generation. Stainless steel provides strong passive corrosion resistance, although localised passive-film breakdown and stress corrosion cracking remain concerns under chloride-rich, elevated-temperature, or microbiologically active conditions. Copper performs favourably under reducing, anoxic repository conditions, but its antimicrobial properties should not be interpreted as immunity to MIC. Sulphide-producing microorganisms can promote CMIC, while contrasting experimental results demonstrate that copper MIC is strongly dependent on environmental conditions and sulphide availability. Titanium exhibits very low general corrosion rates and strong passivity, making it an attractive corrosion-resistant option, but the limited amount of long-term GDF-specific MIC evidence prevents definitive assessment of its behaviour under strongly reducing and microbiologically active conditions. Material selection should therefore remain repository-specific and account for the expected geochemical and microbiological evolution of the complete engineered barrier system rather than relying on a single measure of corrosion resistance.

Three research priorities emerge from this review. First, long-term and in situ experiments should be expanded to determine whether corrosion mechanisms observed in accelerated laboratory studies persist under realistic compacted-buffer and groundwater conditions. Second, experimental methodologies should be standardised to improve comparison between materials, microbial communities, and repository environments. Third, MIC processes should be incorporated more explicitly into long-term corrosion and safety-assessment models, including interactions between microbial metabolism, redox evolution, corrosion products, and passive-film stability. Addressing these priorities will improve the ability to distinguish conservative laboratory observations from credible repository-scale risks and provide a stronger evidence base for material selection and long-term GDF safety assessment.

## Figures and Tables

**Table 1:** Classification of nuclear wastes by activity, half-life, storage technique, and duration, including examples.

Type	Storage Length	Storage Facilities	Examples	Half-Life	Activity
HLW	>100,000 years.	Interim storage for ~100 years due to heat generation, then final disposal in a GDF, 500–1000 m below surface.	Spent nuclear fuel, waste from reprocessing of fuel.	Can exceed 100,000 years.	Typically 10^4^–10^6^ TBq/m^3^; includes any ILW with significant heat generation [8].
ILW	>200 years.	Underground storage, below 20 m. Commonly placed in sections of GDFs (500–1000 m below surface), depending on half-life and activity.	Metal waste from reprocessing nuclear fuel and coolant, reactor decommissioning, and ion-exchange resins [9].	200–500 years	Commonly limited similar to LLW. Can above these limits (>12,000 Bq/g) as it is storage facility dependant [10].
LLW	<200 years.	Underground storage below 10 m or near-surface disposal.	Generated predominantly by nuclear power plants (construction, operation, and decommissioning) but also small quantities from nuclear research, and nuclear medicine [9,10].	100–200 years [11,12]	<12,000 Bq/g of beta activity. <4000 Bq/g alpha activity [13,14].
VLLW	<100 years	Near surface disposal	Waste mainly from decommissioning of nuclear power facilities [9,14].	100 days–100 years [11].	Roughly 1 to 100 Bq/g [9].
VSLW	<2 years.	Near surface disposal	Generated by research and medicine. Solid wastes such as needles, syringes, and protective clothing. Liquid waste is more prominent and is generated by nuclear medical techniques [15].	<100 days [8,9].	
EW	N/A	Landfill.	N/A	N/A	10 μSv or less in a year [8,14].

## Data Availability

No new data were created or analyzed in this study. Data sharing is not applicable to this article.
